# Brain-wide connectivity map of mouse thermosensory cortices

**DOI:** 10.1093/cercor/bhac386

**Published:** 2022-10-18

**Authors:** Phillip Bokiniec, Clarissa J Whitmire, Tobias M Leva, James F A Poulet

**Affiliations:** Max Delbrück Center for Molecular Medicine in the Helmholtz Association (MDC), Berlin, Germany; Neuroscience Research Center, Charité-Universitätsmedizin Berlin, Charitéplatz 1, 10117 Berlin, Germany; Max Delbrück Center for Molecular Medicine in the Helmholtz Association (MDC), Berlin, Germany; Neuroscience Research Center, Charité-Universitätsmedizin Berlin, Charitéplatz 1, 10117 Berlin, Germany; Max Delbrück Center for Molecular Medicine in the Helmholtz Association (MDC), Berlin, Germany; Neuroscience Research Center, Charité-Universitätsmedizin Berlin, Charitéplatz 1, 10117 Berlin, Germany; Institut für Biologie, Humboldt-Universität zu Berlin, Unter den Linden 6, 10099 Berlin, Germany; Max Delbrück Center for Molecular Medicine in the Helmholtz Association (MDC), Berlin, Germany; Neuroscience Research Center, Charité-Universitätsmedizin Berlin, Charitéplatz 1, 10117 Berlin, Germany

**Keywords:** parallel pathways, posterior insular cortex, primary somatosensory cortex, thermosensation, whole-brain connectivity

## Abstract

In the thermal system, skin cooling is represented in the primary somatosensory cortex (S1) and the posterior insular cortex (pIC). Whether S1 and pIC are nodes in anatomically separate or overlapping thermal sensorimotor pathways is unclear, as the brain-wide connectivity of the thermal system has not been mapped. We address this using functionally targeted, dual injections of anterograde viruses or retrograde tracers into the forelimb representation of S1 (fS1) and pIC (fpIC). Our data show that inputs to fS1 and fpIC originate from separate neuronal populations, supporting the existence of parallel input pathways. Outputs from fS1 and fpIC are more widespread than their inputs, sharing a number of cortical and subcortical targets. While, axonal projections were separable, they were more overlapping than the clusters of input cells. In both fS1 and fpIC circuits, there was a high degree of reciprocal connectivity with thalamic and cortical regions, but unidirectional output to the midbrain and hindbrain. Notably, fpIC showed connectivity with regions associated with thermal processing. Together, these data indicate that cutaneous thermal information is routed to the cortex via parallel circuits and is forwarded to overlapping downstream regions for the binding of somatosensory percepts and integration with ongoing behavior.

## Introduction

**Fig. 1:**
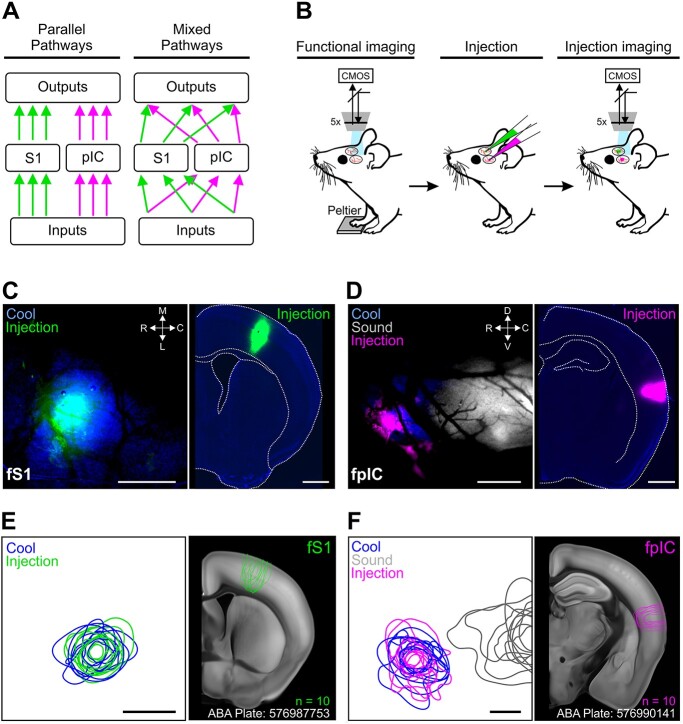
Functional targeting of the thermal representation in fS1 and fpIC. A) Cartoon schematic of parallel and mixed connectivity motifs. B) Schematic representation of the experimental procedure; from left to right, functional identification of the thermal cortical regions using widefield calcium imaging through a cleared skull preparation; injection of different colored retrograde tracers or anterograde viruses; imaging to confirm alignment of injection sites to thermal representation. C) Example mouse showing imaging of fS1 and corresponding coronal brain slice. Left, Overlaid functional response to temperature (blue), with fluorescent tracer (pseudo-colored green); right, post hoc brain slice showing injection site. D) Same as (C), but for fpIC. E) Population injection sites and functional responses of fS1 (*n* = 10 mice). Left shows 80% contours of the widefield thermal response to cool stimuli (blue) and fluorescence of the tracer (green) (*n* = 10 mice, 5 retrograde and 5 anterograde injections) aligned to peak temperature response in fS1. Right shows outlines of all injection sites localized on a coronal brain slice (ABA Plate: 576987753) from the Allen Brain Atlas. F) Left, Same as E (left), but for fpIC and including response to 8 kHz sound stimulation (gray). Right, same as E (right) (ABA Plate: 576990141). Scale bars: C, D, E, F 500 μm.

A fundamental feature of mammalian sensory pathways is that the same modality is represented in multiple cortical areas, but the neuronal wiring principles of multiple cortical sensory representations are unclear. One possibility is that different cortical sensory representations are separate nodes in anatomically segregated, “parallel” neural pathways (Fig. 1A, left). Alternatively, the same presynaptic nuclei could provide copies of sensory information to widespread cortical regions for forwarding to overlapping brain areas in a “mixed” connectivity model (Fig. 1A, right). The thermal system is an ideal model system to address this question as both the primary somatosensory cortex (S1) (Hellon et al. 1973; Tsuboi et al. 1993; Milenkovic et al. 2014) and the posterior insular cortex (pIC) (Penfield and Faulk 1955; Craig et al. 2000; Beukema et al. 2018; Vestergaard et al. 2022) are involved in thermal processing. Moreover, our recent work has shown that both forelimb S1 (fS1) and forelimb pIC (fpIC) play a central role in thermal perception and have a rich cellular representation of cooling (Milenkovic et al. 2014; Vestergaard et al. 2022). However, despite the importance of temperature for somatosensation, there is no comprehensive connectivity map of the mouse thermal system (Bokiniec et al. 2018).

The connectivity of fS1 and IC has been examined independently in prior studies (Guldin and Markowitsch 1983; Cechetto and Saper 1987; McDonald and Jackson 1987; Allen et al. 1991; Shi and Cassell 1998a, 1998b; Kimura et al. 2010; Maffei et al. 2012; Oh et al. 2014; Zakiewicz et al. 2014; Zingg et al. 2014; Gehrlach et al. 2020). Independent tracing allows a comparison of large-scale wiring differences between 2 regions but prohibits a comprehensive examination of connectivity with subregion and cellular resolution. Moreover, most prior studies have used stereotactic targeting for tracer injections which, because blood vessel patterns as well as brain and skull sizes vary from mouse to mouse, makes it difficult to determine whether the cortical injection sites correspond to specific sensory representations.

To examine the connectivity of the thermal representations in fS1 and fpIC, here we targeted tracer injections using functional widefield calcium imaging. Moreover, to allow a direct comparison of input and output wiring of fS1 and fpIC, both regions were injected in the same mice. We used anterograde adeno-associated viruses (AAV) to trace axonal projections (Viswanathan et al. 2015) or cholera toxin subunit B (CTB) for retrograde tracing of cellular resolution inputs. Brains were then sliced and imaged from hindbrain to frontal cortex, and the brain-wide input and output connectivity from thermal representations in fS1 and fpIC were quantified using automated cell counting and axon density estimates. Our study provides a comprehensive whole-brain connectivity map of 2 major thermal cortical representations and suggests that there are independent thermal pathways routed via fS1 and fpIC.

## Materials and methods

### Mice

All experiments were approved by the Berlin Landesamt für Gesundheit und Soziales (LAGeSo) and carried out in accordance with European animal welfare law. Adult (*n* = 10), male and female GP4.3 (C57BL/6J-Tg(Thy1-GCaMP6s)GP4.3Dkim/) mice from Jackson Laboratories (JAX#024275, Chen et al. 2013) were used. Mice were housed under 12-h light/dark cycles and provided with ad libitum food and water.

### Surgery

Mice were anesthetized with isoflurane in oxygen (3%–4% initiation, and 1%–1.5% maintenance, CP-Pharma) and injected with Metamizol for postoperative pain management (200 mg/kg, s.c., Zentiva). Anesthetized mice were then placed in a nose clamp and eye gel (Vidisic, Bausch + Lomb) was applied to both eyes. Core temperature of mice was maintained by a homoeothermic heating blanket (FHC). The right forepaw was tethered onto a Peltier element (8 × 8 mm, Digi-Key Electronics) for thermal stimulation. The left primary somatosensory cortex (S1) was exposed by removing the skin on the parietal bone and locating Bregma and Lambda suture landmarks. The left pIC was exposed by rotating the head ~30 to 40 degrees to the right and displacement of the left temporalis muscle from the temporal bone. The rhinal vein, middle cerebral artery, and zygomatic bone were used as anatomical landmarks for pIC (Vestergaard et al. 2022). The skull overlying S1 and pIC was thinned with a dental drill (head diameter: 0.5 mm, Komet Dental) to improve image quality.

### In vivo imaging

Widefield calcium imaging was used to identify the thermal representations in fS1 and fpIC as previously described (Vestergaard et al. 2022). Briefly, images were acquired by a sCMOS camera (Hamamatsu ORCA-Flash4.O LT) via an epifluorescence stereomicroscope (Excitation: 470/40 nm, Emission: 525/50 nm, Leica MZ10 F) equipped with a CoolLED pE-300 LED Microscope illuminator, at a rate of 20 Hz with 35 ms exposure time. Thermal stimuli were delivered to the right forepaw via a feedback-controlled Peltier element stimulator (custom made device, ESYS GmbH Berlin). Cooling stimuli were 10 or 14 °C drop from 32 °C adapted temperature with a duration of 2 s and onset ramp of 20 °C/s. The location of pIC was further confirmed by identifying the surrounding auditory cortex, and, in some cases, the insular auditory field (see: Rodgers et al. 2008; Sawatari et al. 2011; Gogolla et al. 2014) using an 8 kHz, ~65 dB, 1 s acoustic stimuli delivered via a loudspeaker (Visaton). Mice received a minimum of 3 stimulation trials to confirm functional responses. Craniotomies (~1 × 1 mm) were then performed over the regions responsive to thermal stimulation of the forepaw.

### Tracer and virus injections

Custom written code (Python version 3.7, Python Software Foundation) was used to identify the center point of the widefield response during the imaging session. The center of mass was computed from all pixels above 80% of the peak fluorescence in the trial-averaged responses. Next, glass pipettes (~20 μm diameter) containing cholera toxin subunit B (CTB), for input mapping, or AAV, for axonal output mapping, were inserted into the center of the thermal response, normal to the cortical surface. Two 50–75 nL injections (100 nL/min) were made, one at 700 μm and a second at 400 μm depth from the pial surface, using an oil hydraulic manipulator (One-axis oil hydraulic micromanipulator, Narishige). Pipettes were left in place for 5–10 min following each injection and then slowly retracted. fS1 was injected with either CTB Alexa Fluor 647 (CTB-647, 0.5% in PBS, Thermo Fisher) or AAV-smFP-myc (pAAV.CAG.GFPsm-myc.WPRE.SV40, 7.17 × 10^11^ vg/mL), and fpIC with either CTB Alexa Fluor 555 (CTB-555, 0.5% in PBS, Thermo Fisher) or AAV-smFP-FLAG (pAAV.CAG.Ruby2sm-Flag.WPRE.SV40, 1.58 × 10^12^ vg/ml). To visualize AAV cortical injection sites in vivo, AAVs were mixed with a low concentration of CTB Alexa Fluor 488 (0.05% v/v, 0.5% in PBS, Thermo Fisher).

To confirm that the injection was located in the center of the functional response, we imaged the fluorescence tracer 10 min post injection while on the imaging setup with the same angle, orientation, and field-of-view using either an orange light (excitation: 575/70 nm, emission: 640/50 nm) or a green LED light. A small layer of bone wax was then placed over both craniotomies to prevent tissue damage. The exposed skull was then covered with dental cement (Paladur). Drinking water was supplemented with Metamizol (200 mg/kg, Ratiopharm) for postoperative pain management for 2–3 days.

### Histology

Five to seven days after injection of CTB, or 3–4 weeks after injection of AAV, mice were anesthetized with an overdose of ketamine/xylazine (1,200 mg/kg ketamine, 500 mg/kg xylazine, i.p., WDT eG and Bayer, respectively) and transcardially perfused with 50 mL ice-cold PBS (0.1 M) followed by 50 mL of ice-cold 4% PFA. Brains were removed and post-fixed overnight in PFA at 4 °C. Whole brains were cut into coronal sections (50 μm) using a vibrating microtome (Leica VT1000S) and every fourth section was collected. Sections containing CTB were directly mounted onto glass slides using DAPI Fluoromount-G (Southern Biotech) mounting medium.

Sections containing AAVs were stored for further immunohistochemical processing as described previously (Bokiniec et al. 2017). Briefly, free-floating sections were first washed in PBS containing 0.3% Triton X-100 (3 × 10 min, RT) and then blocked with 5% normal goat serum in the above wash solution for 60 min at RT. Sections were incubated in primary antibodies (diluted in the blocking solution) against myc (rabbit c-Myc, 1:1000, Sigma-Aldrich, C3956, RRID: AB_439680) and FLAG (mouse-FLAG, 1:1000, Sigma-Aldrich, F1804, RRID: AB_262044) for 48 h at 4 °C. Sections were washed with PBS and then incubated in PBS containing 5% normal goat serum with fluorescent conjugated secondary antibodies (Alexa Fluor 555-conjugated goat anti-mouse IgG, 1:500, Thermo Fisher, A21422, RRID: AB_2535844, and Alexa Fluor 647-conjugated donkey anti-rabbit IgG, 1:500, Thermo Fisher, A31573, RRID: AB_2536183) overnight at 4 °C. Brain sections were then washed and mounted onto glass slides using DAPI Fluoromount-G (Southern Biotech) mounting medium.

Brain sections were visualized with a Zeiss upright microscope (Axio Imager A.2) using the ZEN Imaging software. Images were acquired using a 10×/0.45NA objective. Exposure times for AAV or CTB were kept the same across mice.

### Histological image processing

#### Atlas registration

Images were first separated by fluorophore, organized sequentially, rotated to the correct orientation, and downsampled (20% from original) using ZEN Imaging software. Using the ImageJ plugin Fiji (Schindelin et al. 2012), a 1 mm boundary in the rostral–caudal, medial–lateral, and dorsal–ventral axes was masked over the center of the injection sites and excluded from further analysis. All slices were registered to the Allen Brain Atlas Common Coordinate Framework v3 (ABA) using the QUICKNii software package (Puchades et al. 2019). Due to possible section distortion along the dorsal–ventral, rostral–caudal, or medial–lateral axes as a consequence of histological processing, images were adjusted using QUICKNii. Sections were contrast adjusted in QUICKNii to allow clear matching of anatomical landmarks from the slice to the atlas. Following complete registration of the sections to the ABA, the corresponding RGB atlas images were exported from the QUICKNii software.

#### Signal detection

Cell somata were identified using a modified version of AIDAhisto (Pallast et al. 2019) that allows interaction with the ABA RGB atlas (MATLAB Version R2018b, The MathWorks Inc.). Images were filtered using the Leung–Malik Filter Bank (Leung and Malik 2001) to detect noncircular cells with a size between 8 and 10 pixels (corresponding to 20–25 μm in the downsampled image). A single threshold for cell detection was determined empirically and applied to all the datasets. To reduce the identification of false-positive cells, the XY cell positions were referenced to a corresponding binarized DAPI nuclei image using the *k*-nearest neighbor classification where *k* = 1, within a radius of 1.5 pixels. Detected cells were then compared with their corresponding micrographs and any remaining false-positive cells were discarded. ABA RGB coordinates were then obtained by matching the new XY cell positions to the corresponding, transformed RGB atlas image obtained in the Atlas registration step. Finally, we counted the number of cells detected within a region.

Axonal projection density was analyzed with custom-written software (MATLAB Version R2018b, The MathWorks Inc.). Images were first denoised using a Wiener Filter (neighborhood size: 2 × 2). Image slice edges that displayed saturated signal due to histological processing were removed by edge correction from a corresponding binarized DAPI micrograph. Axons were then detected by convolving the images using the Maximum Response 8 (MR8) Gaussian filter bank (Varma and Zisserman, 2005) with a width of 3–6 pixels (corresponding to a minimum and maximum axon width of 1.2 μm and 2.4 μm, respectively, in the downsampled image). A single threshold for axon detection was determined empirically based on the length of the detect axon and was applied to all datasets. The same threshold value was then used for all the corresponding slices and associated datasets. Images were closely matched to the original micrographs to validate axon detection as well as identify and manually remove any residual noise pixels that appeared as a consequence of tissue processing (large, noncontiguous fluorescence). ABA RGB coordinates were then obtained by matching the XY pixel positions with the corresponding transformed RGB atlas image obtained during the Atlas registration step. Finally, we counted the number of pixels detected within a region.

#### Visualization

After atlas registration and signal detection, cell soma (input) and axons (output) were projected onto a 3D reference atlas in Imaris volumetric image software (Version 9.3, Bitplane AG), as previously described for the rat (Dempsey et al. 2017), using the matrix transformations for the ABA described in Puchades et al. 2019.

### Data analysis

Cells and axons were quantified across the whole brain of individual mice using custom-written Python code. Data were normalized as a fraction of the total amount of inputs or outputs detected across the brain. Data were grouped into 6 major brain regions (Cortex, striatum/pallidum, amygdala, thalamus/hypothalamus, midbrain, and hindbrain) with 70 subregions as determined from the ABA. We used the terms fS1 and fpIC for the 2 injection sites. For all analysis of inputs and outputs, we used the subregion terms listed in Supplementary Table 1.

Input–output Pearson correlation coefficients were performed for each major brain region (cortex, thalamus, and amygdala). Independent *t*-tests for each brain region were performed on the percentage of whole-brain inputs or outputs between fS1 and fpIC. All values are expressed as mean ± SEM unless otherwise stated. Differences were considered statistically significant at *P* < 0.05.

To visualize the spatial alignment of the functional response and the injection location (Fig. 1E and F—left), fluorescence contours were aligned across mice using custom-written Python code as described previously (Vestergaard et al. 2022). Briefly, the functional fluorescence images (cool- and sound-trial average evoked responses) and the anatomical fluorescence image (injection location) were smoothed with a Gaussian filter (σ = 20 pixels). The center of mass was computed from all pixels above a threshold of 80% of peak fluorescence for the trial-averaged cool-evoked responses to identify the center of the cortical region sensitive to temperature. For visualization of the functional signal, 80th percentile contours for each field of view (fS1, fpIC) from individual mice were translated to align to the center of mass of the fluorescence for thermal stimulation. For anatomical fluorescence images, the contours were superimposed on the corresponding atlas section (Fig. 1E and F—right).

To assess the spatial separability of the inputs or outputs within a given subdivision of the brain, the point cloud of coordinates for inputs (cell somata) and outputs (axons) within each subdivision was converted into a mesh in the ABA coordinate space. Contralateral coordinates were removed for this analysis. To minimize sampling limitations due to tissue thickness, the mesh was smoothed (spatial Gaussian, standard deviation = 100 μm). The mesh was converted to a binary matrix at a threshold value of one-tenth of the maximum voxel. A binary matrix was generated for fS1 inputs, fS1 outputs, fpIC inputs, and fpIC outputs for each brain subdivision. An overlap parameter was estimated for each subdivision as:

$$\begin{align*} &{\text{Overlap}}_{\text{input}}\\&=\frac{\sum \text{fS1}_{\text{binary},\text{input}}\ \text{AND}\ {\text{fpIC}}_{\text{binary},\text{input}}}{\sum \text{fS1}_{\text{binary},\text{input}}\!+\!\sum{\text{fpIC}}_{\text{binary},\text{input}}-\sum \text{fS1}_{\text{binary},\text{input}}\ \text{AND}\ {\text{fpIC}}_{\text{binary},\text{input}}} \end{align*}$$

$$\begin{align*} &{\text{Overlap}}_{\text{output}} \\ &=\!\frac{\sum \text{fS1}_{\text{binary},\text{output}}\ \text{AND}\ {\text{fpIC}}_{\text{binary},\text{output}}}{\sum\! \text{fS1}_{\text{binary},\text{output}}\!+\!\sum\! {\text{fpIC}}_{\text{binary},\text{output}}\!-\!\sum\! \text{fS1}_{\text{binary},\text{output}}\ \text{AND}\ {\text{fpIC}}_{\text{binary},\text{output}}} \end{align*}$$

Random data sets (*n* = 50 per subdivision) were generated under the null hypothesis by shuffling the labeling of each coordinate included in the analysis for Monte Carlo hypothesis testing. The *P* value was computed as the proportion of simulated overlap coefficients greater than the observed overlap coefficient.

To visualize the spatial overlap, the mesh was not binarized. Instead, a 3-dimensional contour plot was generated (isosurface, Matlab) at the 30th quantile of the non-zero voxels. As shown in Fig. 6F and G, this spatial map was generated across 3 thalamic nuclei: VPL, PO, and PoT.

### Data exclusion

Data were excluded if: the injection site was not located in the cortical functional response; post hoc examination showed that the injection site was mistargeted; the retrograde injection spread into the underlying white matter tract (corpus callosum).

## Results

### Functionally targeted tracer injections into fS1 and fpIC

We targeted the thermosensitive regions of fS1 and fpIC using wide-field calcium imaging in anesthetized GP 4.3 mice (Fig. 1B). A cooling stimulus (32–22 °C) was delivered to the glabrous skin of the forepaw and evoked responses were visualized online (Fig. 1C and D, left). The location of forelimb thermal representation in pIC was further confirmed by functional identification of the neighboring auditory cortex using an auditory tone (Fig. 1D and F, left). A small craniotomy was performed, and anatomical tracers (CTB or AAV) were injected into the center of the functional response (Fig. 1B). Finally, the spatial overlap between the functional response and injection was confirmed with in vivo imaging (Fig. 1E and F, left).

Seven days after injection of CTB, or 3–4 weeks after injection of AAVs, mice were perfused and brains removed for histological processing. Analysis of the CTB and AAV injection sites in fS1 and fpIC showed that the spread of the tracers in the injection site was not significantly different and spread throughout the entire cortical column (medial/lateral fpIC: 565 ± 40 μm, fS1: 653 ± 77 μm; dorsal/ventral fpIC: 927 ± 46 μm, fS1: 986 ± 50 μm; rostral/caudal fpIC: 800 ± 55 μm, fS1: 800 ± 43 μm) (Fig. 1E and F; Supplementary Fig. 1). To identify brain-wide input and output nuclei, fluorescent images of the whole brain were taken and registered to the Allen Brain Atlas Common Coordinate Framework v3 (ABA, Supplementary Fig. 3). Regions 1 mm rostral/caudal and dorsal/ventral (parallel with the cortical region) from the center of the injection site were excluded from further analysis due to saturation of the fluorescent signal. DAPI staining of cell bodies and comparison to a cortical slice from the Scnn1a-Tg3-Cre mouse, which selectively expresses Cre-recombinase in cortical layer 4 neurons (JAX#009613, Madisen et al. 2010), crossed with a tdTomato-expressing Cre-reporter mouse (Ai9, JAX#007909, Madisen et al. 2010), suggests that the injections were targeted to a putative granular region of pIC (putative layer 4 thickness fS1: 205 ± 14 μm and fpIC: 215 ± 11 μm, Supplementary Fig. 2).

Whole-brain input–output connectivity maps were created from coronal sections spaced 200 μm apart from +1.4 to −7.0 mm relative to bregma. Analysis of the olfactory bulbs, frontal cortical regions and the cerebellum were excluded. As the number of labeled neurons (fS1: 5147 ± 800, fpIC: 7737 ± 330 cells, *n* = 5 mice) and axons (fS1: 2,323,883 ± 245,388, fpIC: 1,862,505 ± 286,668 pixels, *n* = 5 mice) varied across mice, the input and output values were normalized as a fraction of the total amount of input cell bodies/axonal outputs detected across the entire brain.

### Whole-brain input–output connectivity of fS1 and fpIC

**Fig. 2:**
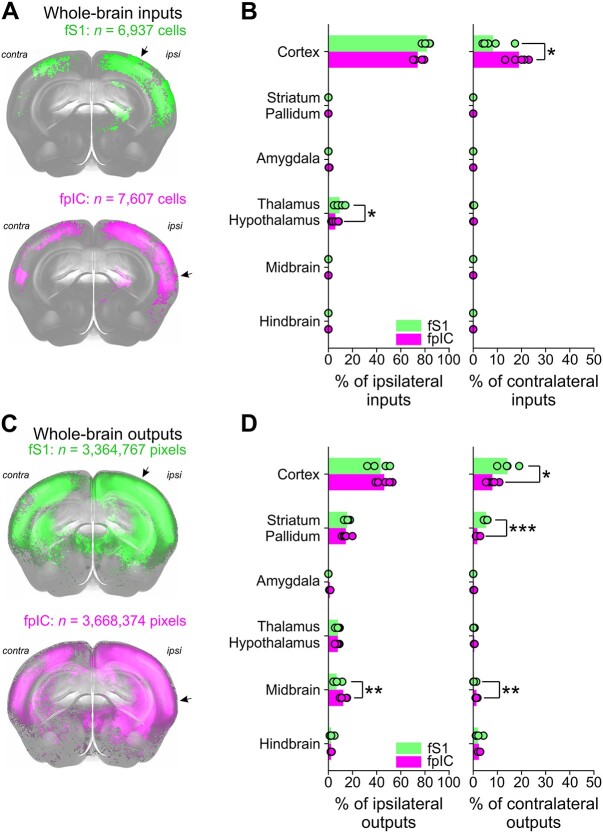
Whole-brain long-range inputs and outputs of fS1 and fpIC. A) Front view of an example 3D brain reconstruction showing brain-wide cell bodies providing input to fS1 (top—green, *n* = 6,937 identified cell bodies) or fpIC (bottom—magenta, *n* = 7,607 identified cell bodies). Arrows indicate injection site. B, A comparison of the ipsilateral (left) and contralateral (right) inputs from 6 major brain regions to fS1 (green) or fpIC (magenta) as a percentage of the whole-brain inputs. Bars show means and circles show data from individual mice. ^*^P < 0.05, *n* = 5 mice per condition. C) Same as B but showing reconstruction of pixels labeled with axonal outputs (green, *n* = 3,364,767 pixels; magenta *n* = 3,668,373 pixels). D) Same as B, but showing a comparison of cortical axonal outputs in target regions. ^*^P < 0.05, ^*^^*^P < 0.01, ^*^^*^^*^P < 0.001, *n* = 5 mice per condition.

To quantify the whole-brain inputs and outputs of fS1 and fpIC at a broad scale (Fig. 2A and C), we first divided the data into 6 major regions: cortex, striatum/pallidum, amygdala, thalamus/hypothalamus, midbrain, and hindbrain (Fig. 2B and D). The majority of inputs originated in the side ipsilateral to the injection site, with contralateral inputs almost exclusively located in the contralateral cortex (Fig. 2B, fS1 ipsi: 82%, fS1 contra: 8%, fpIC ipsi: 74%, fpIC contra: 19%). The overall brain-wide distribution of inputs was similar for fS1 and fpIC, with the cortex being the dominant source of ipsilateral inputs to fS1 and fpIC; however fpIC showed a higher proportion of inputs from contralateral cortical regions than fS1 (Fig. 2B—right, fS1: 8%, fpIC: 19%, *P* = 0.022, *n* = 5 mice). The ipsilateral thalamus was the second major input region, with significantly more inputs projecting to fS1 than fpIC (Fig. 2B—left, fS1: 9%, fpIC: 6%, *P* = 0.013, *n* = 5 mice). Intriguingly, the amygdala projected to fpIC, but not to fS1 (Fig. 2B, left).

As for the inputs to fS1 and fpIC, brain-wide outputs mostly targeted regions ipsilateral to the injection site (Fig. 2D, fS1 ipsi 74%, fS1 contra 26%; fpIC ipsi 86%, fpIC contra 14%). The major target of fS1 and fpIC axons was the cortex, which received a similar amount of ipsilateral innervation (Fig. 2D—right, fS1 43%, fpIC 46%, *P* = 0.111, *n* = 5 mice), whereas projections from fS1 to the contralateral cortex were stronger than projections from fpIC (Fig. 2D—left, fS1 14%, fpIC 8%, *P* = 0.013, *n* = 5 mice). The second major innervation target of fS1 and fpIC was the striatum/pallidum, which received similar levels of ipsilateral input (Fig. 2D—left, fS1 15%, fpIC 14%, *P* = 0.626, *n* = 5 mice), but significantly more contralateral input from fS1 than fpIC (Fig. 2D—right, fS1 5%, fpIC 2%, *P* = 0.001, *n* = 5 mice). Ipsilateral and contralateral axonal targets from fS1 and fpIC innervated the thalamus (Fig. 2D, fS1 ipsi 8%, fpIC ipsi 8%, *P* = 0.982, *n* = 5 mice; fS1 contra 0.2%, fpIC contra 0.4%, *P* = 0.193, *n* = 5 mice) and hindbrain (Fig. 2D, fS1 ipsi 2%, fpIC ipsi 2%, *P* = 0.988, *n* = 5 mice; fS1 contra 2%, fpIC contra 2%, *P* = 0.526, *n* = 5 mice) to similar amounts. Both ipsilateral and contralateral sides of the midbrain received more innervation from fpIC compared to fS1 (fS1 ipsi 7%, fpIC ipsi 12%, *P* = 0.004, *n* = 5 mice; fS1 contra 0.3%, fpIC contra 1%, *P* = 0.002, *n* = 5 mice). Closely resembling the inputs, the amygdala was innervated exclusively by fpIC outputs (fpIC ipsi 1.2%, fpIC contra 0.2%) and not by fS1.

To examine connectivity at higher resolution, we went on to subdivide the 6 major anatomical areas into 70 subregions and present data from regions ipsilateral to the injection side. For a comprehensive list of all ipsilateral and contralateral connections, see Supplementary Figs. 4 and 5 and Supplementary Tables 2 and 3. To assess fS1 and fpIC connectivity at different scales, we compare the connectivity strength as well as spatial distributions of regions, subregion nuclei, and single cells.

### Whole-brain subregion inputs to fS1 and fpIC

**Fig. 3:**
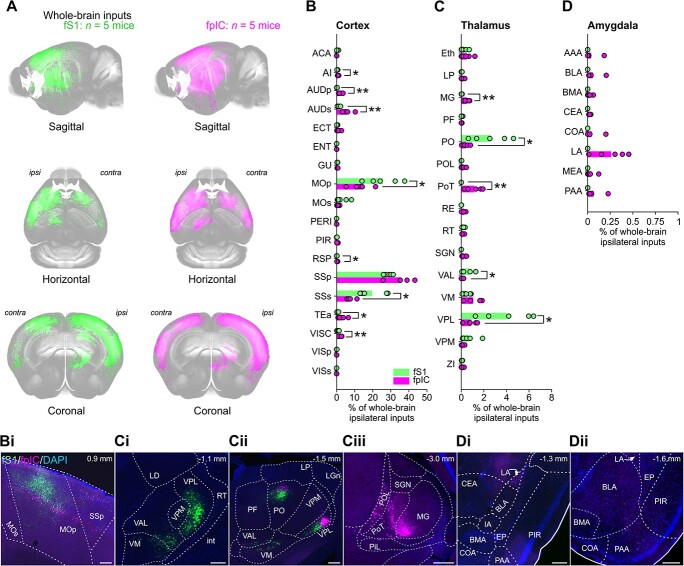
Whole-brain ipsilateral inputs to fS1 and fpIC. A) Whole-brain 3D image of cell bodies labeled with CTB-647 (pseudo-colored green, fS1 injection) or CTB-555 (pseudo-colored magenta, fpIC injection) (*n* = 5 mice). Cell bodies were identified and registered to the Allen CCF v 3.0. B–D) Proportions of whole-brain ipsilateral inputs to fS1 (green) or fpIC (magenta) from (B) cortical, (C) thalamic, or (D) amygdaloid subregions. Bars show means and circles show individual mice. ^*^P < 0.05, ^*^^*^P < 0.01, *n* = 5 mice per condition. See Supplementary Table 2 for values for all subregions. Representative example brain slices of inputs to fS1 (green) or fpIC (magenta) from selected (Bi) cortical, (Ci, Cii, Ciii) thalamic, or (Di, Dii) amygdaloid subregions. The full list of abbreviations is shown in Supplementary Table 1. Scale bars: Bi, Ci, Cii, Ciii, Di, Dii 250 μm.

Visualizing inputs to fS1 and fpIC at different angles of a 3D projection revealed dense labelling across many cortical and thalamic nuclei (Fig. 3A, Supplementary Table 2, Supplementary Movie 1). The majority of inputs showed a similar innervation strength to fS1 and fpIC. Notable exceptions included stronger inputs to fS1 than fpIC from regions involved in sensorimotor processing, including primary motor cortex (MOp, fS1 26%, fpIC 13%, *P* = 0.035, *n* = 5 mice, Fig. 3B and Bi) and supplemental somatosensory cortex (SSs, fS1 20%, fpIC 8%, *P* = 0.010, *n* = 5 mice). In support of its role in diverse sensory and cognitive functions (Gogolla 2017), fpIC received more input than fS1 from a broader range of cortical nuclei, including agranular insular cortex (AI, fpIC 0.8%, fS1 0.35%, *P* = 0.019, *n* = 5 mice), primary and supplemental auditory cortices (AUDp, fpIC, 2.5%; fS1 0.16%, *P* = 0.002, *n* = 5 mice; AUDs, fpIC 5.4%, fS1 0.97%, *P* = 0.009, *n* = 5 mice), retrosplenial cortex (RSP, fpIC 0.4%, fS1 0.14%, *P* = 0.029, *n* = 5 mice), temporal association area (TEA, fpIC 3.15%, fS1 0.74%, *P* = 0.046, *n* = 5 mice), and visceral cortex (VISC, fpIC 2.1%, fS1 0.62%, *P* = 0.004, *n* = 5 mice). fS1 received significantly more thalamic input from nuclei within the ventral basal and posterior thalamic compartments (Fig. 3C, Ci, and Cii), including the ventral posterolateral (VPL, fS1 4.1%, fpIC 0.87%, *P* = 0.014, *n* = 5 mice), the ventral anterolateral (VAL, fS1 0.72%, fpIC 0.07, *P* = 0.039, *n* = 5 mice), and posterior medial (PO, fS1 2.6%, fpIC 0.41%, *P* = 0.020, *n* = 5 mice, Fig. 3Cii) regions. Thalamic innervation of the fpIC was more diverse than fS1, with significantly more input from the medial geniculate nucleus (MG, fpIC 0.5%, fS1 0.03%, *P* = 0.002, *n* = 5 mice) and a prominent innervation from the primary triangular (PoT) nucleus (Fig. 3Ciii, fpIC 1.2%) that did not project to fS1. In agreement with prior literature (Shi and Cassell 1998a, 1998b; Schiff et al. 2018), we did not observe any innervation of fS1 by the amygdala (Fig. 3D). In contrast, fpIC was innervated by cortical-like regions of the amygdala, the majority of which came from the lateral amygdala (LA, 0.3%, Fig. 3Di). The basolateral (BLA, 0.06%, Fig. 3Dii) and piriform-amygdala area (PAA, 0.07%, Fig. 3Dii) innervated fpIC to a lesser extent and very few retrogradely labeled cell bodies from fS1 and fpIC were observed in the striatum-like centromedial nuclei (CEA, MEA both <0.05%). While at a gross scale, we observed similarities in inputs to fS1 and fpIC (Fig. 2), differences start to emerge at subregion level (Fig. 3).

### Whole-brain subregion output targets of fS1 and fpIC

**Fig. 4:**
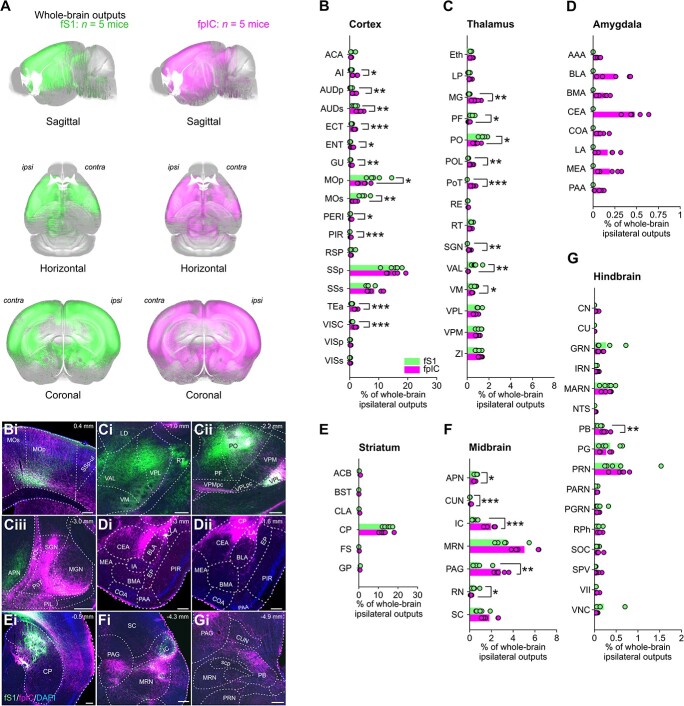
Whole-brain ipsilateral outputs from fS1 and fpIC. A) AAVs expressing fluorescent proteins were injected into the thermally responsive areas of fS1 and fpIC following widefield imaging. Axons projecting from fS1 (left) or fpIC (right) were extracted and registered to the Allen CCF v 3.0) (*n* = 5 mice). Proportions of whole-brain ipsilateral outputs from fS1 (green) or fpIC (magenta) to (B) cortical, (C) thalamic, (D) amygdaloid, (E) striatal, (F) midbrain, or (G) hindbrain subregions. Bars show means and open circles show individual mice. ^*^P < 0.05, ^*^^*^P < 0.01, *n* = 5 mice per condition. See Supplementary Table 3 for values of all subregions. Representative example brain slices of outputs from fS1 (green) or fpIC (magenta) from different (Bi) cortical, (Ci, Cii, Ciii) thalamic, (Di, Dii) amygdaloid, (Ei) striatal, (Fi) midbrain, and (Gi) hindbrain subregions. Scale bars: Bi, Ci, Cii, Ciii, Di, Dii, Ei, Fi, Gi 250 μm.

Axonal projections from fS1 and fpIC broadly innervate many regions of the mouse brain (Fig. 4A), with fS1 and fpIC both having more output than input regions. At subregion resolution, we noted a number of significant differences in the comparative strengths of innervation (Fig. 4B–F, Supplementary Table 3, Supplementary Movie 2). Similar to the pattern of inputs to fS1 and fpIC, axonal outputs from fS1 strongly innervated regions involved in sensorimotor processing, such as primary and secondary motor cortices (MOp, fS1 9%, MOs, 5%, Fig. 4Bi), and fpIC innervated a broader number of cortical regions than fS1, including AI (fpIC 1.6%, fS1 0.45%, *P* = 0.019, *n* = 5 mice), AUDp (fpIC 1.6%, fS1 0.4%, *P* = 0.005, *n* = 5 mice), AUDs (fpIC 3.5%, fS1 1.8%, *P* = 0.009, *n* = 5 mice), ectorhinal (ECT, fpIC 1.6%, fS1 0.7%, *P* = 0.0004, *n* = 5 mice), entorhinal (ENT, fpIC 0.8%, fS1 0.18%, *P* = 0.025, *n* = 5 mice), gustatory (GUS, fpIC 0.7%, fS1 0.22%, *P* = 0.004, *n* = 5 mice), piriform (PIR, fpIC 0.4%, fS1 0.06%, *P* < 0.0006, *n* = 5 mice), perirhinal (PERI, fpIC 0.5%, fS1 0.22%, *P* = 0.026, *n* = 5 mice), TEa (fpIC 2%, fS1 0.71%, *P* = 0.002, *n* = 5 mice), and VISC (fpIC 1.9%, fS1 0.89%, *P* = 0.001, *n* = 5 mice). Notable differences between the innervation of the thalamus by fS1 and fpIC included significantly more outputs from fS1 to the ventral anterior-lateral complex (fS1 VAL 0.7%, fpIC 0.09%, *P* = 0.003, *n* = 5 mice, Fig. 4Ci), ventral medial nucleus (VM, fS1 0.7%, fpIC 0.46%, *P* = 0.044, *n* = 5 mice, Fig. 4Ci), parafascicular nucleus (PF, fS1 0.5%, fpIC 0.18%, *P* = 0.025, *n* = 5 mice, Fig. 4Cii), and PO (fS1 1.5%, fpIC 0.89%, *P* = 0.013, *n* = 5 mice, Fig. 4Cii); whereas fpIC more strongly innervated MG (fpIC 0.8%, fS1 0.2%, *P* = 0.004, *n* = 5 mice), the suprageniculate nucleus (SGN, fpIC 0.2%, fS1 0.01%, *P* = 0.003, *n* = 5 mice), and the posterior limiting nucleus (POL, fpIC 0.4%, fS1 0.08%, *P* = 0.002, *n* = 5 mice). As with their inputs, the PoT was selectively innervated by fpIC and not by fS1 (0.6%, Fig. 4Ciii) and the amygdala was innervated by fpIC and not by fS1 (Fig. 4D). The majority of fpIC outputs to the amygdala targeted the striatum like central and medial amygdala nuclei (CEA and MEA, collectively 0.67%, Fig. 4Di and Dii) and, to a lesser extent, the cortical like basolateral and basomedial nuclei (BLA, BMA, and LA, collectively 0.54%, Fig. 4Di and Dii). The major striatal target of both fS1 and fpIC was the caudate putamen (CP, Fig. 4E and Ei). Outputs from fS1 and fpIC targeted a range of nuclei in the midbrain (Fig. 4F) and hindbrain (Fig. 4G). The primary midbrain target of fS1 and fpIC was the midbrain reticular nucleus (MRN, Fig. 4Fi). fS1 axons innervated the anterior pretectal nucleus (APN, 0.62%, fpIC 0.45%, *P* = 0.04, *n* = 5 mice, Fig. 4Ciii) and the red nucleus (RN, 0.44%, fpIC 0.13%, *P* = 0.042, *n* = 5 mice) more than fpIC. fpIC showed significantly more innervation of the cuneiform nucleus (CUN, fpIC 0.12%, fS1 0.03%, *P* < 0.001, *n* = 5 mice), inferior colliculus (IC, fpIC 2%, fS1 0.43%, *P* < 0.001, *n* = 5 mice, Fig. 4Fi), and the periaqueductal gray (PAG, fpIC 2.8%, fS1 0.85%, *P* = 0.001, *n* = 5 mice, Fig. 4Fi) than fS1. Hindbrain subregions received similar levels of axonal output from fS1 and fpIC (Fig. 4G), with one notable difference being the stronger innervation of the parabrachial nucleus by the fpIC (PB, fpIC 0.25%, fS1 0.1%, *P* = 0.006, *n* = 5 mice, Fig. 4Gi), an area that forwards thermal information to circuits in the hypothalamus that regulate body temperature.

### Brain-wide reciprocal connectivity of fS1 and fpIC

**Fig. 5:**
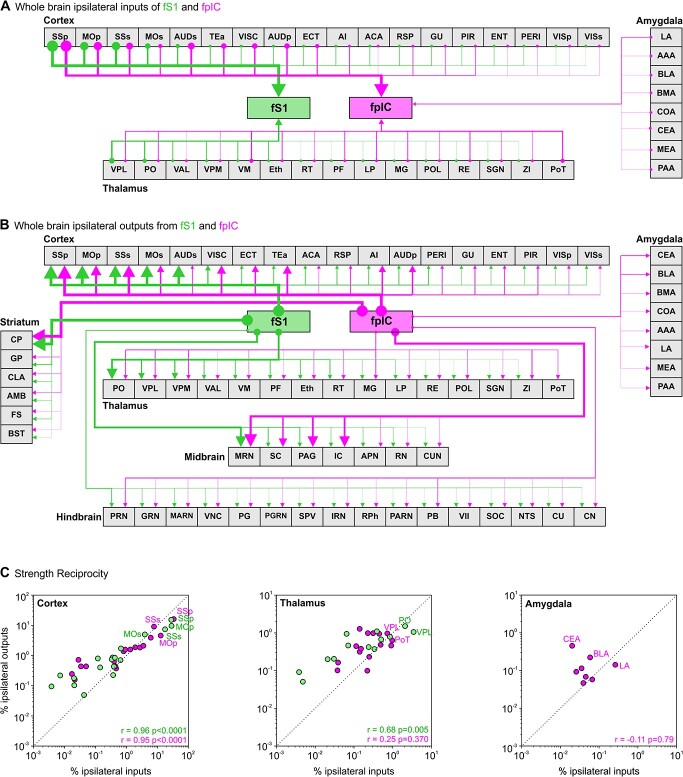
Brain-wide wiring diagram and reciprocal connectivity. A) Schematic brain-wide wiring diagram showing projections to fS1 (green) or fpIC (magenta). Regions are ordered from left to right based on the strength of their projections to fS1 with the line thickness proportional to the strength of inputs in Fig. 3 and Supplementary Table 2. A list of abbreviations is provided in Supplementary Table 1. B) Same as (A) but for axonal projections. C) Reciprocal connectivity of inputs and outputs of fS1 and fpIC. Graphs show correlations of the strength of input/outputs for fS1 (green) and fpIC (magenta) for subregions in (left) cortex, (middle) thalamus, and (right) amygdala. Individual data points correspond to the mean value of a subregion (*n* = 5 mice, *r* = Pearson’s correlation coefficient), all subregions named in Supplementary Fig. 6.

A canonical feature of cortical wiring is reciprocal connectivity, whereby regions providing input also receive outputs from the target region. Plotting the input and output circuit diagrams from our tracing data highlighted that a number of cortical and thalamic regions were reciprocally connected with fS1 and fpIC (Fig. 5A and B). In contrast, the hindbrain, midbrain, and striatum only received axonal projections from fS1 and fpIC without providing direct inputs. To investigate the reciprocal connectivity of fS1 and fpIC with brain-wide subregions further, we plotted the strengths of cortical, thalamic, and amygdaloid inputs against their respective innervation from fS1 and fpIC (Fig. 5C, Supplementary Fig. 7). In agreement with an established model of cortico-cortical connectivity (Felleman and Van Essen 1991), we observed strong reciprocity for both fS1 and fpIC with other cortical regions (fS1 *r* = 0.96, *P* < 0.0001, fpIC *r* = 0.95, *P* < 0.0001). As expected (Hunnicutt et al., 2014), the thalamus was also highly reciprocally connected with fS1 (r = 0.68, *P* = 0.005). The thalamic and amygdala nuclei targeted by fpIC axons also provided reciprocal input to the same nuclei (Fig. 5C). The strength of connectivity, however, was dominated by outputs; therefore, the correlation between the normalized strength of input versus output connectivity was not significant at the population level (amygdala, *r* = −0.11, *P* = 0.79; thalamus, *r* = 0.25, *P* = 0.370).

### Spatial organization of fS1 and fpIC whole-brain inputs and outputs

**Fig. 6:**
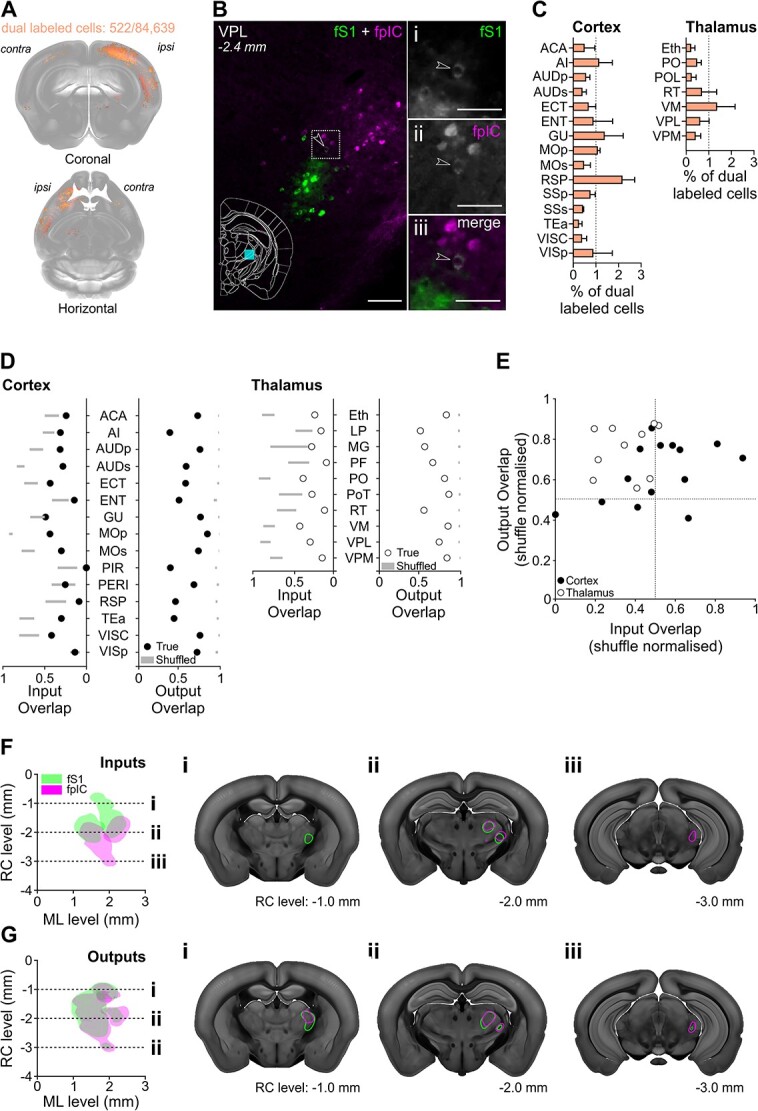
Spatial organization of fS1 and fpIC cortical and thalamic inputs and outputs. A) Cell bodies labeled with both CTB-647 and CTB-555 (pseudo-colored orange) that project to fS1 and fpIC registered to the Allen CCF v 3.0 (*n* = 5 mice). B) Representative micrograph of a coronal brain section showing the VPL nucleus with CTB positive cells projecting to fS1 (i) or fpIC (ii), and one identified cell (iii - white, highlighted by arrowhead) that projects to both fS1 (green) and fpIC (magenta). C, Percentage of dual labeled cells projecting to fS1 and fpIC in cortical and thalamic nuclei (mean +/− SEM, *n* = 5 mice). D) Input and output overlap coefficients quantified from multiple cortical or thalamic nuclei. Circles show mean true data while gray bars represent 99% confidence intervals of the shuffled distributions. E) Input and output overlap coefficients normalized by the shuffle distribution mean for each region. Each circle shows an individual brain region, with open circles for thalamic regions and filled circles for cortical regions. Labelling of individual data points is provided in Supplementary Fig. 7. The position of the data points in the upper left quadrant of the graph demonstrate that inputs are less overlapped than outputs. Note that thalamic regions (open circles) are further from the diagonal than cortical regions (black), indicating that thethalamic regions show a greater difference in their input/output spatial overlap than cortex. F, Representation of reconstructed VPL, PO, and PoT inputs to fS1 (green) or fpIC (magenta) (*n* = 5 mice). Left, Plotted as a horizontal view (rostrocaudal vs. mediolateral). Right shows coronal sections highlighting (i) a rostral region with input to fS1 only, (ii) an intermediate rostrocaudal region with input to both fS1 and fpIC, and (iii) a caudal region with inputs to fpIC only. G) Same as F but for outputs to thalamus from fS1 and fpIC. Scale bars: B, left 100 μm, Bi, Bii, Biii 50 μm.

At the subregion level, fS1 and fpIC have some similar input structures and output targets. However, this does not address whether there was target specific connectivity of individual cells (parallel pathways) or an absence of distinct substructure organization (mixed pathways). To assess whether subregions contained cells projecting to both fS1 and fpIC, cells labeled with both retrograde tracers were counted and projected onto the 3D mouse brain. Dual labeled neurons were sparse (Fig. 6A) compared to the total inputs innervating fS1 or fpIC and from the 84,639 total cells that projected to fS1 or fpIC (*n* = 5 mice), we identified only 522 that were dual labeled (Fig. 6A, Bi, and Bii). In the cortical and thalamic subregions providing input to both fS1 and fpIC (Fig. 6C), only 0.65% of all cortical and 0.36% of all thalamic neurons projected to both fS1 and fpIC. Together, these data indicate that sensory input to fS1 and fpIC is provided by separate, parallel, circuits.

As the inputs to fS1 and fpIC arose primarily from separate populations of neurons, we went on to analyze the spatial organization of clusters of inputs within each cortical and thalamic subregion. We defined spatial separability, or overlap, as the percent of voxels containing both fS1 and fpIC projecting neurons within a given subregion (see Methods). A completely separable map-like organization would have an overlap value of 0, while a completely intermingled salt-and-pepper like organization would have an overlap value of 1. We found that within the cortex and thalamus, most co-labeled subregions showed nonrandom, spatially organized inputs that were significantly different from their shuffled distributions (Fig. 6D). This indicates that inputs from the cortex and thalamus to fS1 and fpIC are organized in a spatially separate map-like arrangement within individual subregions.

We next asked whether this was also true for the spatial distribution of axonal projections from fS1 and fpIC. We found that cortical and thalamic regions had less spatial overlap of fS1 and fpIC axons than expected from a random distribution (Fig. 6D). However, while both inputs and outputs demonstrated spatial separability, the separation for inputs is higher than outputs (average input overlap 0.25, average output overlap 0.67, Fig. 6D). Even after normalizing the overlap value to the shuffled control value, the input overlap is lower than the output overlap across cortical and thalamic subregions (Fig. 6E, Supplementary Fig. 8). This can be visualized most clearly in thalamic regions where there is a clear rostrocaudal division in both the input and output volumes (Fig. 6F). In a horizontal projection of VPL, PO, and PoT, the fS1 inputs are spatially localized to the rostral region of thalamus, while the fpIC inputs are spatially localized to the caudal regions. At more rostral levels, coronal slices contain exclusively fS1 projecting cells (Fig. 6Fi), while caudal coronal slices contain exclusively fpIC projecting cells (Fig. 6Fiii) with some overlap at intermediate rostrocaudal levels (Fig. 6Fii). In the corresponding output plots, there is a significantly different distribution of fS1 and fpIC axonal outputs, but a reduced separability compared to thalamic inputs (Fig. 6G).

While fS1 and fpIC share multiple input and output nuclei at a gross scale (Fig. 2), these results suggest that there are more specific patterns of connectivity within cortical and thalamic subregions. The input neurons projecting to fS1 and fpIC arise from spatially separate populations within each subregion, supporting the hypothesis that fS1 and fpIC have parallel input pathways. Though the output projections of fS1 and fpIC showed nonrandom spatial separability within each subregion, this separation was lower than the input populations, supporting a more mixed model of fS1 and fpIC outputs.

## Discussion

Here we used functionally targeted tracer injections to generate a comprehensive map of long-range inputs and outputs from 2 cortical representations of temperature. This approach allowed a direct comparison of fS1 and fpIC connectivity in the same mice. While both areas receive input from common cortical and thalamic regions, fpIC thalamic input is more widespread and, at cellular resolution, inputs to both areas originated from largely nonoverlapping and spatially separated neuronal populations. Despite receiving independent inputs, fS1 and fpIC innervate similar long-range cortical and subcortical regions with axonal projections that were less spatially separated than their inputs, implying that the formation of coherent thermal percepts involves the convergence of cortical outputs. Notably, exclusive connectivity was observed between the fpIC and the amygdala, PB of the hindbrain, and the PoT. Together, our data suggest that thermal information forms at least 2 separate pathways to fS1 and fpIC which then widely broadcast thermal information across the brain.

### Identification and nomenclature of pIC

A classic approach to address wiring of a brain region is to inject neuronal tracers using bregma coordinates for targeting a region of interest. Bregma coordinates, however, are notoriously variable from mouse to mouse. To address this, we functionally targeted our injections to the center of the widefield cortical calcium response to cool stimuli delivered to the forepaw. In order to standardize connectivity maps between mice, we then aligned brain slices to the mouse brain atlas from the Allen Mouse Brain Common Coordinate Framework version 3, which is widely used to standardize maps of neural circuitry (ABA, Pollak Dorocic et al. 2014; Do et al. 2016; Niedworok et al. 2016; Zhang et al. 2016; Fürth et al. 2018; Wang et al. 2020; Benavidez et al. 2021; Dempsey et al. 2021). Aligning to the ABA allowed correction for multiplane distortion, misalignment, and tissue deformation during tissue processing; however, area borders in the ABA have chiefly been constructed using anatomical markers rather than functional properties of regions, potentially leading to discrepancies in less well-studied areas.

In this study, functionally targeted injections into the thermal region of fS1 were localized post hoc in the primary somatosensory upper limb region of the ABA (SSp-ul). Injections into the forepaw thermal region of pIC, however, were labeled as supplemental somatosensory cortex (SSs) or the supplemental auditory area (AUDs, see Fig. 1). Work in multiple mammalian species has identified a somatotopic representation of cutaneous tactile input located in an area ventral to supplemental somatosensory cortex (SSs), dorsal to the rhinal vein, bordering rostral regions of the auditory cortex and containing an anterior auditory field and termed parietal ventral (PV) area or pIC (in squirrels: Krubitzer et al. 1986, hedgehog: Catania et al. 2000, opossum: Beck et al. 1996, marmosets/macaques: Krubitzer and Kaas 1990; Krubitzer et al. 1995, mice: Gogolla et al. 2014; Nishimura et al. 2015, rat: Fabri and Burton 1991a; Remple et al. 2003; Rodgers et al. 2008; Zhang et al. 2020). Using calcium imaging, we have recently shown that this area contains somatotopically organized, rich cellular representation of cool and warm (Vestergaard et al. 2022), in contrast to the cool dominated representation in fS1. Together with its profound impact on thermal perception, these data support the hypotheses that this region houses the primary cortical representation of temperature.

Recently, Gămănuţ et al. (2018) generated a new horizontal cortical map using multiple histological staining methods including transgenic mice expressing fluorophores (tdTomato) in parvalbumin neurons and neurons expressing the muscarinic acetylcholine receptor, as well as cytochrome oxidase and VGlut2 staining. In this map, the borders of the visceral cortex (VISC), a cortical region that may be homologous to granular IC, are significantly different to that of the ABA (see Supplementary Figs. 1 and 3 of Gămănuţ et al. 2018), with VISC forming a lip that envelopes SSs and extends to the dorsal auditory area (AUDd) rather than ending abruptly and giving rise to the temporal association area (TEa) and ventral auditory area (AUDv) as in the ABA. In our recent work (see Supplementary Fig. 1 of Vestergaard et al. 2022), alignment of flattened cortical sections to the horizontal cortical atlas modified from Gămănuţ et al. (2018) showed that the thermally responsive region in pIC was localized in VISC and agrees well with their separation of the SSs boundary with VISC. Comparing our injection site locations to coronal sections from the Scnn1a-cre mouse line, which labels layer 4 cortical neurons (Madisen et al. 2010), suggested that pIC is localized within a granular region of IC (Supplementary Fig. 2).

While prior work has named this area PV or pIC, given their tightly overlapping location and similar sensory representation, we support the proposal by Rodgers et al. (2008) and Nishimura et al. (2015) that PV and pIC are in fact homologous areas. In agreement with the naming used by prior work in rodents (Rodgers et al. 2008; Gogolla et al. 2014; Beukema et al. 2018; Zhang et al. 2020) and evidence of thermal information processing in human pIC (Craig et al. 2000), in this study and in Vestergaard et al. (2022), we use the term pIC. To resolve this issue further and confirm the borders between AUDv, VISC, and SSs, future work should perform detailed functional somatotopic mapping of thermal and tactile responses in pIC and SSs using acoustic stimuli to mark the insular auditory field and AUDv and AUDp followed by post hoc histological mapping.

### Neuronal tracing

A critical factor in the interpretation of tracing data lies in the functional specificity of the labeling method. Our core motivation was to map and compare S1 and pIC circuits involved in cool processing using functionally targeted injections into the forepaw regions of S1 and pIC. However, fS1 is also responsive to tactile stimulation of the skin and pIC encodes warm and cool and contains tactile and auditory subregions (the insular auditory field) (Sawatari et al. 2011; Vestergaard et al. 2022). CTB and AAV tracing therefore could have labeled touch, temperature and, in pIC, possibly, auditory responsive cells. One way to better understand the specificity of the wiring of the cool pathway further could be to compare our data to injections made in subregions of S1 or pIC without thermal responsiveness. However, because the cool sensitive transient receptor potential cation channel subfamily melastatin member 8 (TRPM8) is expressed in primary sensory afferent neurons innervating the entire skin surface, including skin between the whisker follicles (Dhaka et al. 2008), widespread regions of S1 and pIC likely show thermally responsivity. Moreover, while widefield imaging of fpIC shows a separation of the peak tactile and thermal response, warm and cool cells are spatially intermingled, and in fS1, there is spatial overlap of tactile and cool responsive zones (Milenkovic et al. 2014; Vestergaard et al. 2022). Therefore, this study provides a first blueprint for the wiring of cool responsive cortical regions, but in future studies it will be important to link wiring to functional tuning with cellular resolution. One approach could be to use sensory evoked activity-dependent expression of fluorescent proteins (e.g. Beukema et al. 2018) or methods like CANE or TRAP (Capturing and manipulating Activate Neural Ensembles, and Targeted Recombination in Active Populations) coupled with anterograde or retrograde tracers (Guenthner et al. 2013; Sakurai et al. 2016; Wall et al. 2019), while another could be visually targeted single-cell electroporation of functionally identified cells (Wickersham et al. 2007; Marshel et al. 2010; Rancz et al. 2011). Given the differences in the cortical encoding and perception of warm and cool (Vestergaard et al. 2022), we predict that the wiring of thermally responsive cortical neurons will be dependent on their thermal tuning properties.

Our study shared limitations common to anatomical tracing studies (Saleeba et al. 2019). First, analysis of local connectivity was prevented because of the saturated fluorescence CTB and AAV labelling ~1mm^3^from the injection site. We therefore masked this region from analysis. Second, though the direction of transport of CTB is primarily retrograde, it can also label in an anterograde direction. In our datasets, and only at high illumination for signals close to saturation, we observed some anterograde CTB+ve axons innervating the striatum (data not shown), but as our automated input analysis was tuned to identify cell soma (see Methods), these axons were left undetected and discarded upon manual confirmation. Third, CTB can be taken up by fibers of passage rather than terminating axons or cell bodies (Chen and Aston-Jones 1995). The use of transsynaptic retrograde tracing strategies, including rabies virus-based tracing (Wickersham et al. 2007), retrograde AAVs (Tervo et al. 2016), or herpes simplex virus 1 (HSV-1, Ugolini et al. 1987), may help address this issue in future studies. However, these methods have their own caveats including tropism for cortical layers and cell types, and the cellular mechanisms of transsynaptic transport remain unclear. Moreover, due to the high connectivity between S1 and pIC, transsynaptic rabies tracing would likely label both fS1 and fpIC making it difficult to identify the starter population during simultaneous injections. Lastly, while AAV-based labelling is highly effective in labelling axonal projections, it does not specifically label synaptic boutons and therefore analysis of axonal projections will include fibers of passage as well as terminating fibers. Future use of tools that label presynaptic sites, e.g. synaptophysin-cre-based viruses (Beier et al. 2015; Lerner et al. 2015; Knowland et al. 2017; Dempsey et al. 2021) could help resolve this.

### Comparison to prior anatomical tracing studies

Our data show similar overall connectivity to previous mapping studies from rodent fS1 (Fabri and Burton 1991a, 1991b; Zingg et al. 2014). For example, we observed that inputs to fS1 originated from cortical somatosensory (SSp, SSs) and motor regions (MOp) as well as lemniscal and paralemniscal thalamic nuclei (VPL, PO). Moreover, fS1 targeted similar cortical, striatal, thalamic, midbrain, and hindbrain subregions to the whole-brain fS1 output mapping by Zakiewicz et al. 2014. Two differences to Zakiewicz et al. (2014) were strong projections from fS1 to PAG and MRN and the lack of innervation of the substantia nigra. These differences could result from the use of different model systems (rat vs. mouse), different tracers (BDA vs. AAV), or injection volumes and spread. In support of this, a similar approach using virally expressed tracers by Oh et al. (2014) showed axonal projections from forelimb fS1 to PAG and MRN, whereas only faint projections were observed following anterograde tracer injections of *Phaseolus vulgaris* leuco-agglutinin (Pha-L) or biotinylated dextran amine (BDA) in Zingg et al. (2014).

The connectivity of rodent insular cortex has received great attention (Akers and Killackey 1978; Guldin and Markowitsch 1983; Cechetto and Saper 1987; Allen et al. 1991; Shi and Cassell 1998a, 1998b; Kimura et al. 2010; Mathiasen et al. 2015; Gehrlach et al. 2020), but in the majority of these studies tracer injections were not functionally targeted and only partially labeled the thermal fpIC region we examine here. Detailed comparisons are therefore challenging, but, in broad agreement with these studies, we found that somatosensory and associated motor cortices (SSp, SSs, and MOp) as well as key somatosensory thalamic nuclei (VPL, PO, PoT) and amygdaloid subregions (LA, PAA, and BLA) provide input to fpIC. Likewise, output targets were similar to those reported by Shi and Cassell (1998a) and Kimura et al. (2010) who targeted the rat insular auditory field using electrophysiological mapping. One difference to Kimura et al. (2010) was the projection from fpIC to the midbrain PAG; however, in agreement with our data, lateral PAG innervation has been observed from a caudal granular insular cortex region in mice (Oh et al. 2014; Zingg et al. 2014).

### Functional implications

An understanding of cortical sensory processing requires detailed knowledge of the sensory input driving cortical responses. While our recent work has highlighted the cellular encoding of non-painful thermal information in the cortex (Vestergaard et al. 2022), the thalamic representation of temperature is not well understood and has typically been investigated in the context of thermal pain. Prior work in humans and nonhuman primates has suggested that cool sensation might be encoded by a posterior region of the ventral medial nucleus (VMpo) (Craig et al. 1994; Davis et al. 1999). Studies in anesthetized rodents have observed cool responses in the ventral basal complex (including VPL, Hellon and Misra 1973; Schingnitz and Werner 1980) and noxious heat in the PoT (Gauriau 2004). Moreover, anatomical work has shown that PoT is strongly innervated by spinal lamina I/II neurons (Gauriau and Bernard 2004), together leading to the proposal that it is the rodent homologue of VMpo (Gauriau 2004). Our data show that VPL and PO innervate fS1 and fpIC, whereas PoT is connected to fpIC but not fS1 (Figs. 3 and 4). The identification of different cellular encoding schemes in fS1 and fpIC (Vestergaard et al. 2022) alongside the differences of thalamic connectivity to fS1 and fpIC (Figs. 3 and 4) suggest that different thalamic nuclei may show specific thermal response properties. Future work should compare the cellular encoding of thermal information across the thalamic structures identified here and link their activity to thermal perception.

An intriguing aspect of the thermal system is its link to valence. That the thermal system can evoke pain is well appreciated, but non-painful thermal stimuli can also trigger context-dependent pleasant or unpleasant sensations. For example, skin cooling can be pleasurable if the environmental or body temperature is hot, but unpleasant if cool (Chatonnet and Cabanac 1965; Cabanac et al. 1972). The amygdala plays a central role in the encoding of emotion and valence (Sah et al. 2003; Gründemann et al. 2019) and recently has been implicated in the integration of external sensory information with internal states (Gehrlach et al. 2019; Dolensek et al. 2020; Livneh et al. 2020; Livneh and Andermann 2021). Here we show that the thermal fpIC is strongly connected with the amygdala (Fig. 5), and one possibility is that this pathway is involved in the link between thermosensation, internal state, and valence.

Alongside the perception of external environmental temperature, the thermal system plays a fundamental role in the regulation of internal body temperature. External thermal information is forwarded from the sensory periphery via the parabrachial nucleus (PB) to hypothalamic circuits which regulate body temperature (Tan and Knight 2018; Madden and Morrison 2019). Manipulation of the activity of PB has shown that it plays a key role both in body temperature regulation and the coordination of thermoregulatory behaviors like locomotion to thermal regions (Morrison and Nakamura 2011; Yahiro et al. 2017; Morrison and Nakamura 2019). How the perceptual system and the thermoregulatory systems interact is unclear, but our data indicate that there is a projection from fpIC to the PB (Fig. 4) which could provide a top-down modulation of PB. Future work combining PB recordings with manipulation of this pathway could address this hypothesis.

## Outlook

Understanding the wiring of a sensory system is required for a mechanistic understanding of its function. The thermal system has been understudied compared to many other sensory pathways, but recent behavioral and neural data have shown that it is a fast, sensitive, and robust system with a profound impact on perception, valence, and innate behaviors. We hope that the connectivity maps identified here will provide a springboard for future investigations into the diverse and fundamental functions of the thermal system.

## Funding

This work was supported by the European Research Council (ERC-2015-CoG-682422, JFAP), the Deutsche Forschungsgemeinschaft (DFG, FOR 2143, JFAP; SFB 1315, JFAP), and the Helmholtz Society (JFAP). CJW was supported by Human Frontier Science Program (HFSP LT000359/2018-L).

*Conflict of interest statement*: The authors declare no competing interests.

## Data and material availability

Datasets and code are available upon reasonable request.

## Author contributions

PB and JFAP designed the study. PB performed all experiments. PB, CJW, and TML analyzed the data. PB and JFAP wrote the manuscript with comments from all authors.

## Supplementary Material

Bokiniec_SupplementaryInformation_bhac386

Bokiniec_SuppMovie1_bhac386

Bokiniec_SuppMovie2_bhac386
