# Supplementary material for: Brain-wide connectivity map of mouse thermosensory cortices
Source: Cereb Cortex. 2022 Oct 18;33(8):4870–85. doi: 10.1093/cercor/bhac386 (PMC10110442; doi:10.1093/cercor/bhac386)
Supplement: Bokiniec_SupplementaryInformation_bhac386 [file bokiniec_supplementaryinformation_bhac386.zip › Bokiniec_SupplementaryInformation_bhac386.pdf]

Supplementary Information

Brain-wide connectivity map of mouse thermosensory cortices

Phillip Bokiniec, Clarissa J. Whitmire, Tobias M. Leva, and James F. A. Poulet

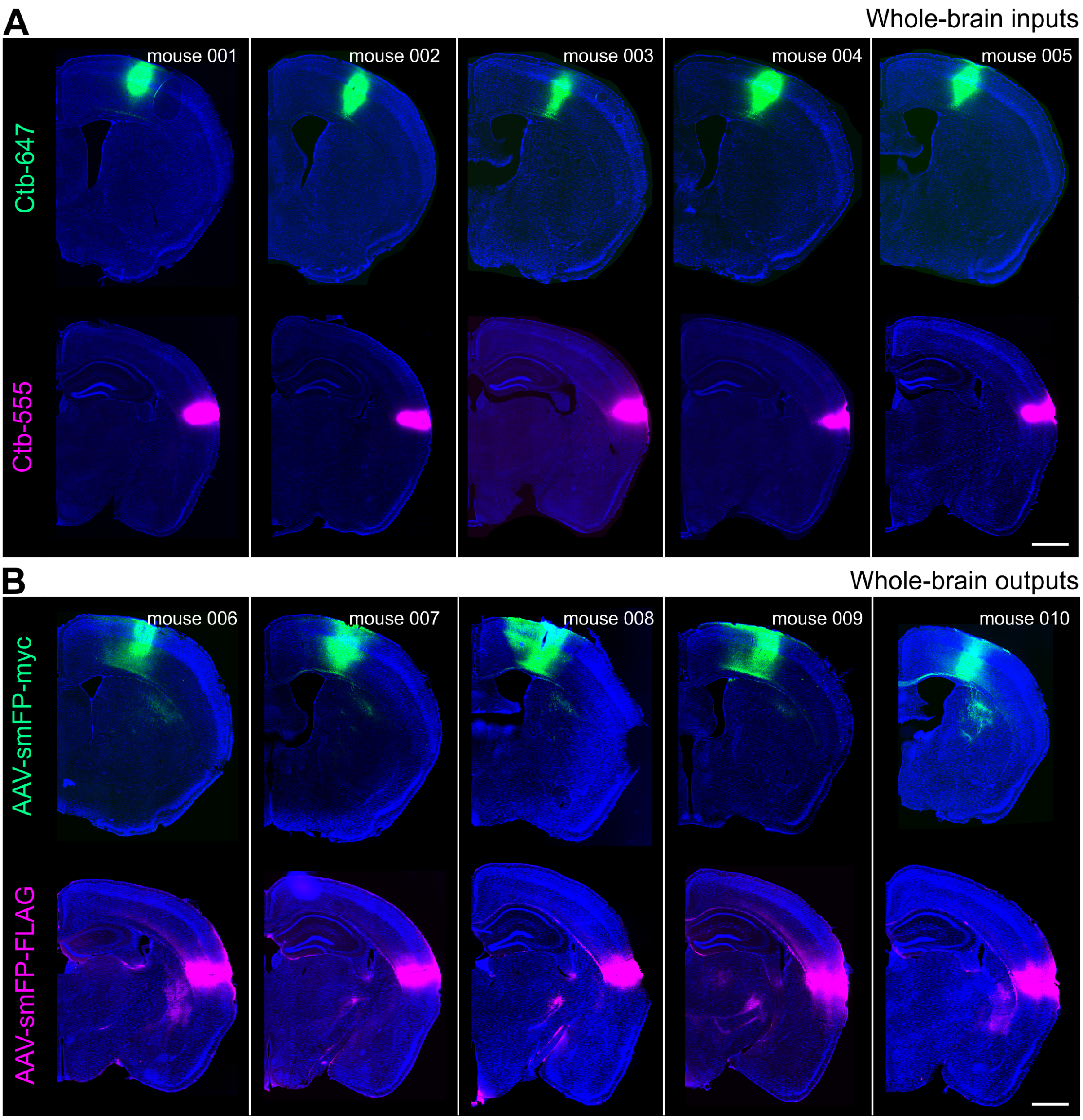

Supplementary Figure 1. Example brain slices showing injection sites from all mice in study.

A, CTB injection sites into thermal representation in fS1 (green, top) or fpIC (magenta, bottom) for input mapping. **B**, As in **A** except for injection of AAV tracers for output mapping. Scale bars **A, B** 1 mm.

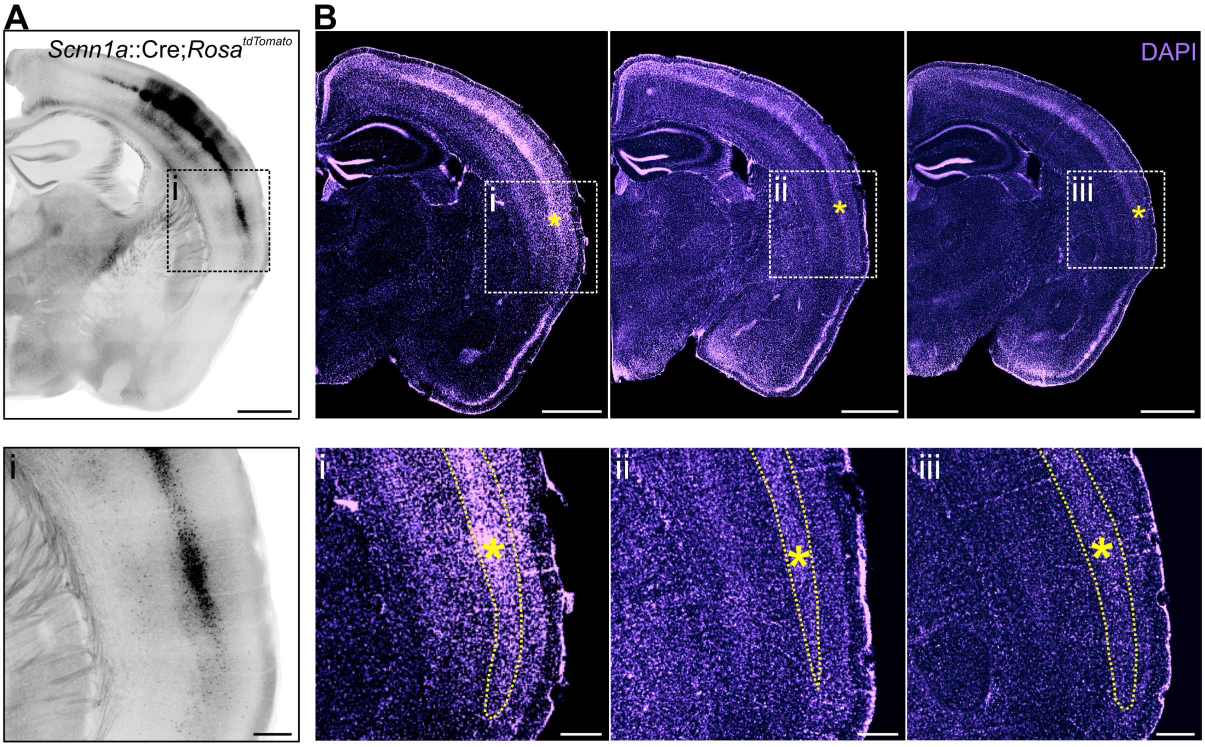

Supplementary Figure 2. Putative granular layer 4 in fpIC

A, Top, representative micrograph of a coronal brain slice at the estimated rostro-caudal slice of thermal fpIC in mouse line expressing tdT in layer 4 neurons (Scnn1a-Tg3-Cre mouse crossed with the tdTomato reporter Ai9 mouse). Bottom, higher resolution image of the same slice with a granular layer (black cell bodies) in the putative thermal responsive region (see also Supplementary Figure 1 of Vestergaard et al., 2022). **B**, Representative micrographs of DAPI staining from 3 example mice in the tracing dataset. Boxes show estimated region with dense granular layer comparable to that seen in **Ai**. Yellow asterisk indicates the center of injection site following widefield calcium imaging, yellow dashed line represents estimated border of granular layer 4. Scale bars, **A, B – top** 1 mm, **Ai, Bi Bii, Biii** 250 µm.

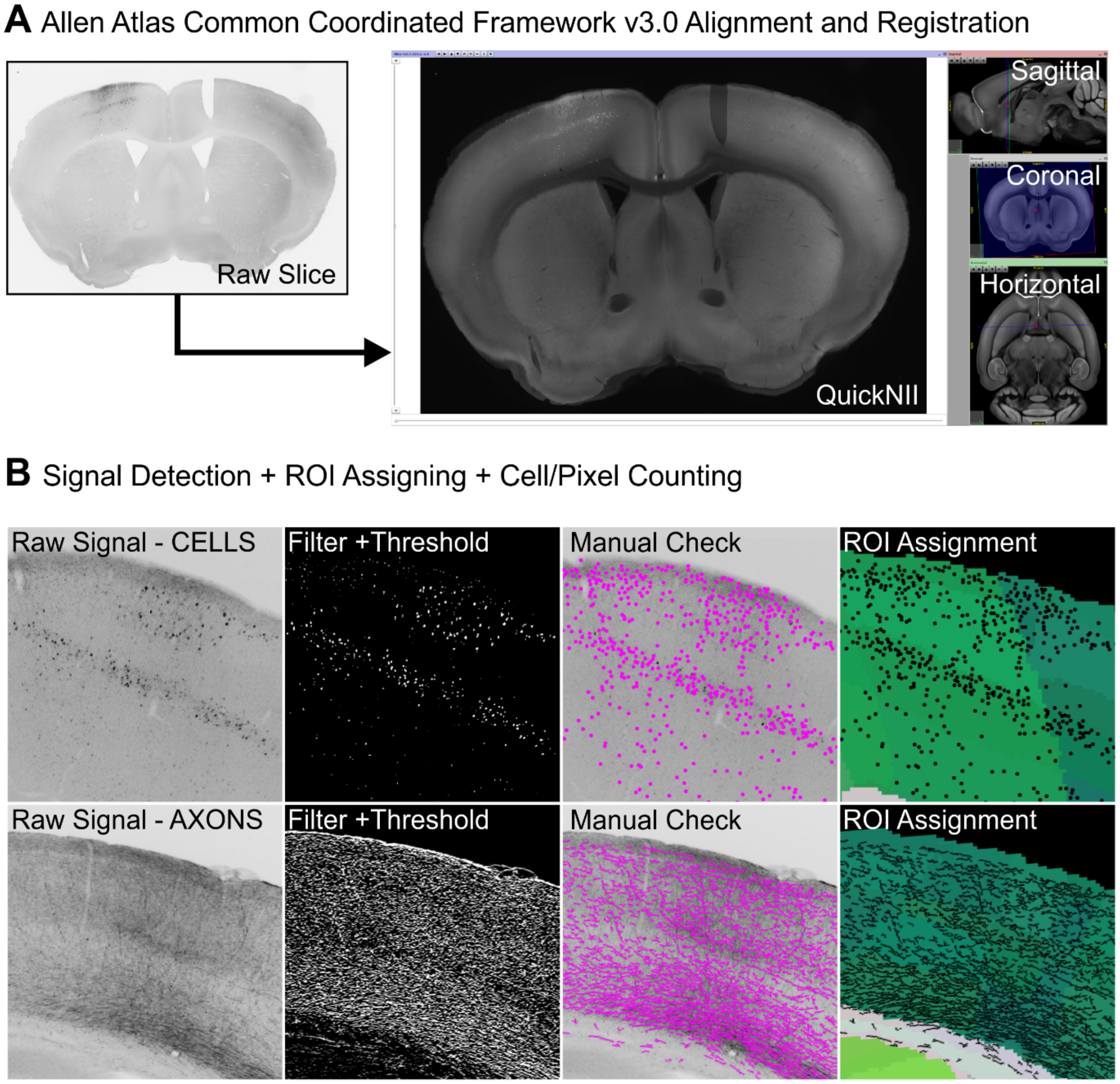

Supplementary Figure 3. Analysis sequence to quantify whole-brain input/outputs.

A, Brain sections were aligned and registered to the Allen Atlas Common Coordinated Framework v3.0 (Wang et al., 2020) using the QUICKNii Software (Puchades et al., 2019). **B**, Top, example fpIC slice showing signal filtering, thresholding, manual verification and ROI assignment of single cell bodies providing input. Bottom, same as top except for axonal output data.

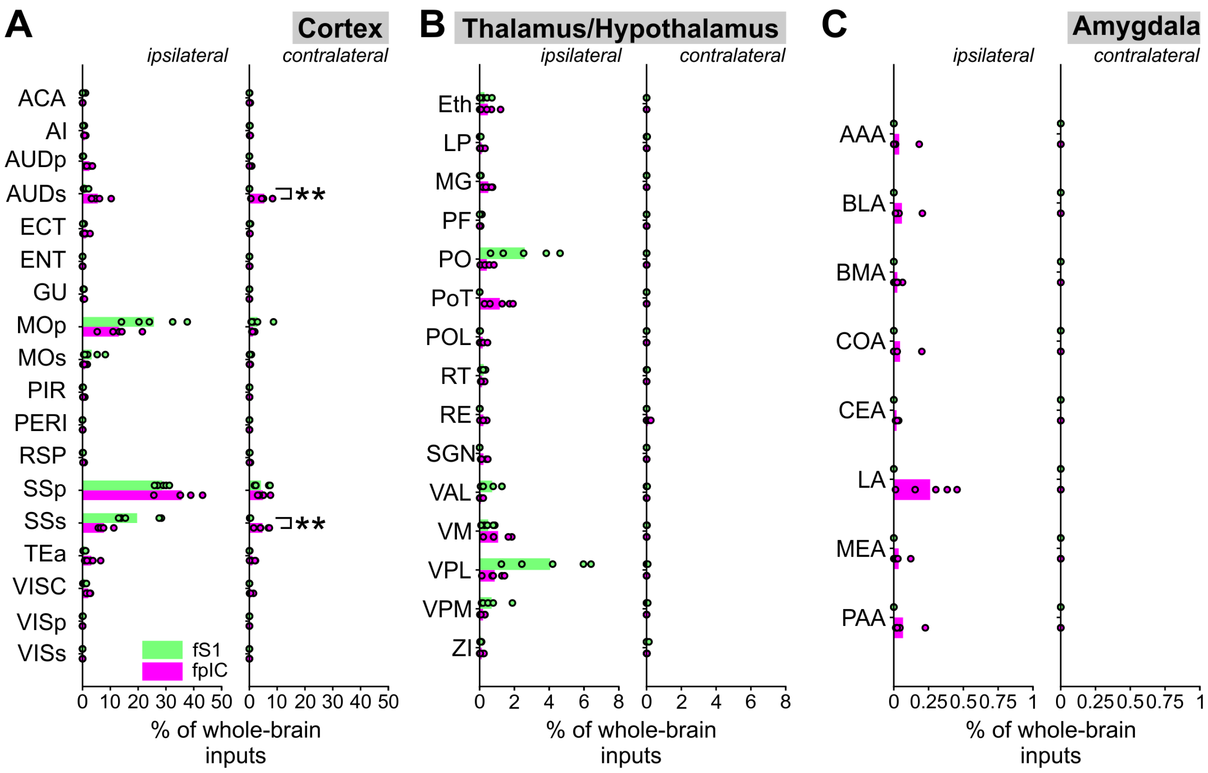

Supplementary Figure 4. Ipsilateral and contralateral inputs to thermal cortices. Comparison of ipsilateral (left) with contralateral (right) (**A**) cortical, (**B**) thalamic/hypothalamic, and (**C**) amygdaloid inputs to fS1 (green) and fpIC (magenta). Bars show means and circles show individual mice, *n* = 5 mice per condition. * = *P* < 0.05, ** = *P* < 0.01, *** = *P* < 0.001. For *P* values, see Supplementary Table 2. A full list of abbreviations is provided in Supplementary Table 1.

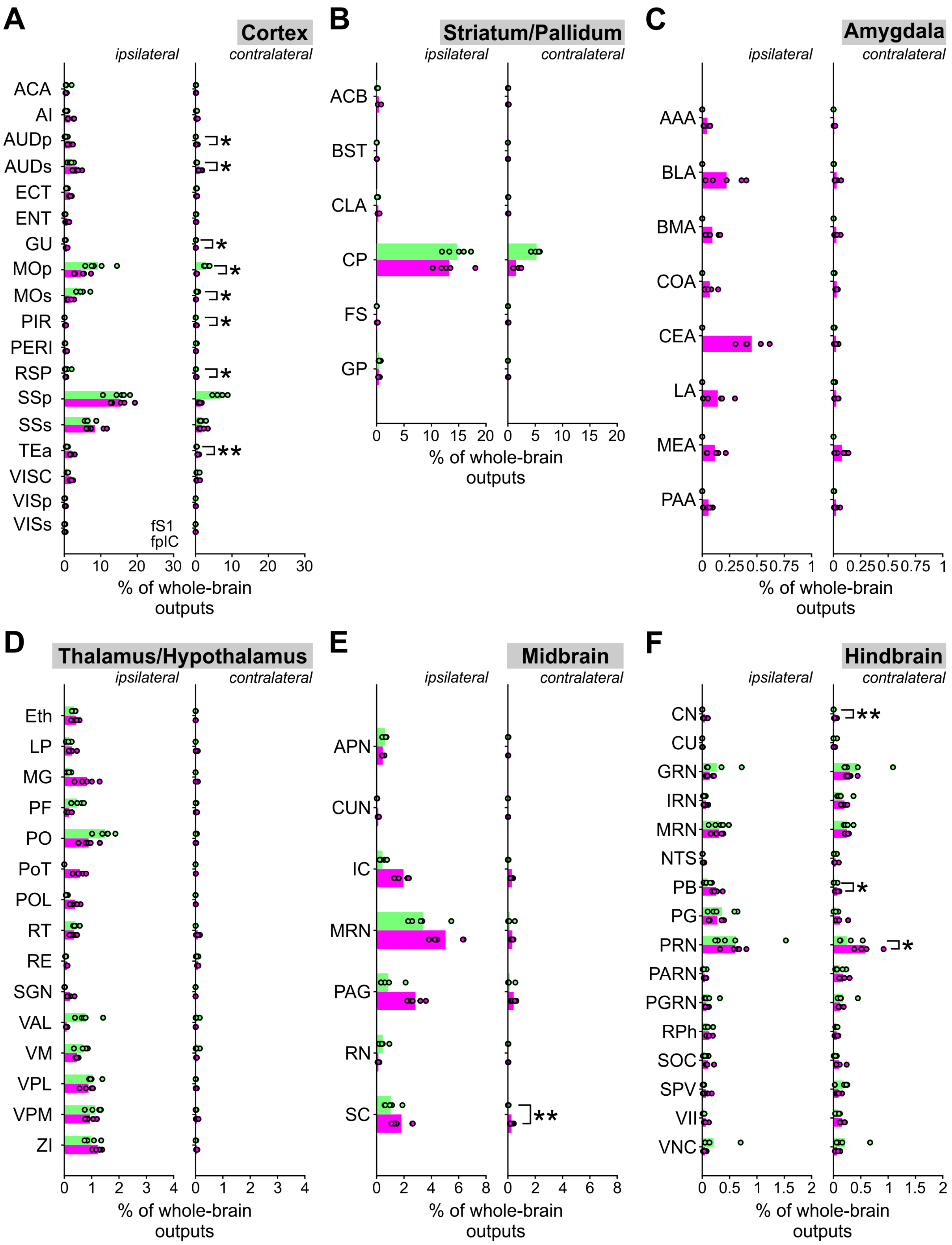

Supplementary Figure 5. Ipsilateral and contralateral outputs from thermal cortices. Comparison of fS1 (green) and fpIC (magenta) ipsilateral (left) or contralateral (right) outputs to subregions of the (**A**) cortex, (**B**) striatum/pallidum, (**C**) amygdala, (**D**) thalamus/hypothalamus, (**E**) midbrain, and (**F**) hindbrain. Bars show mean value and open circles show individual mice, $n = 5$ mice per condition. * = $P < 0.05$, ** = $P < 0.01$, *** = $P <$

0.001. For *P* values, see Supplementary Table 3. A full list of abbreviations is provided in Supplementary Table 1.

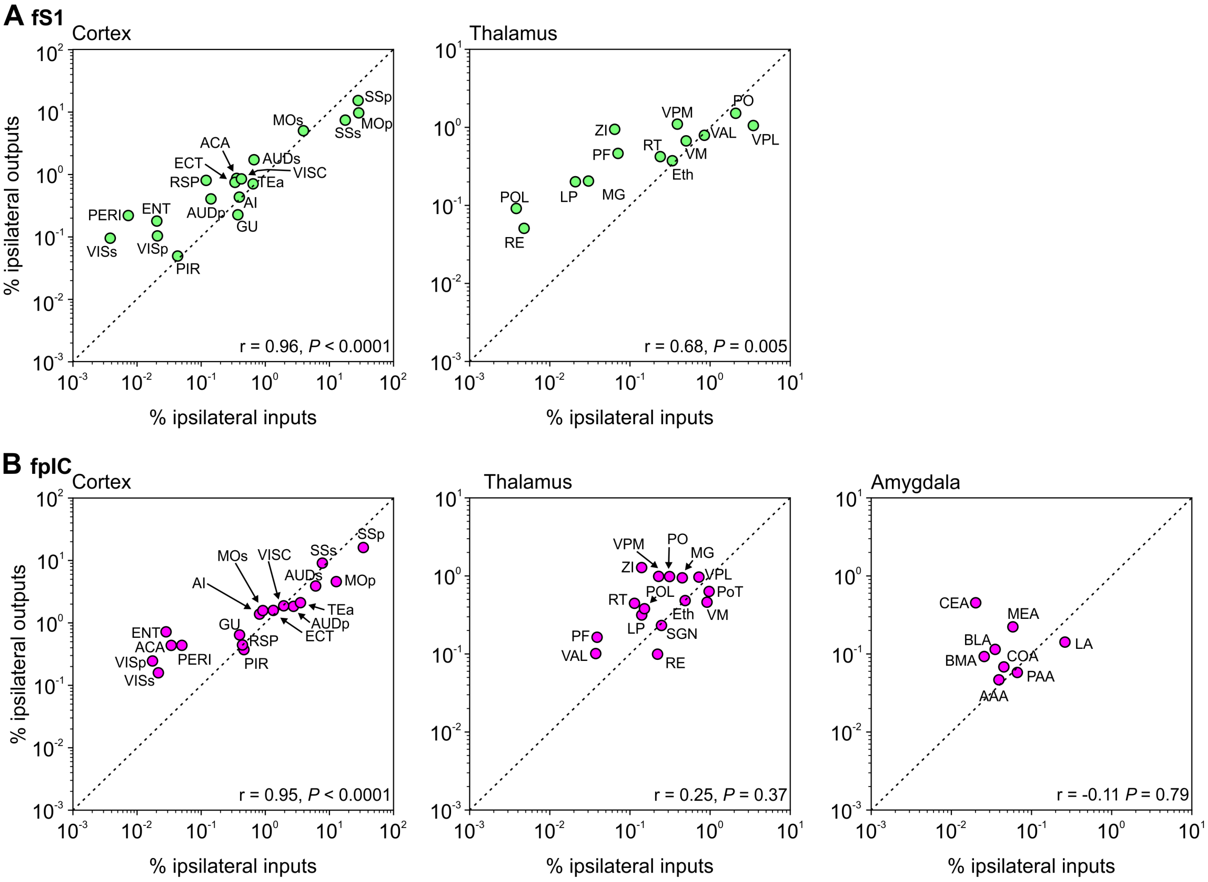

Supplementary Figure 6. Correlations of input-output strength for fS1 and fpIC.

A, Correlations of input-output strength for cortical and thalamic subregions connected to fS1. Individual data points show mean value. r = Pearson's correlation coefficient. **B**, Same as (**A**), but for fpIC and including connectivity with Amygdala.

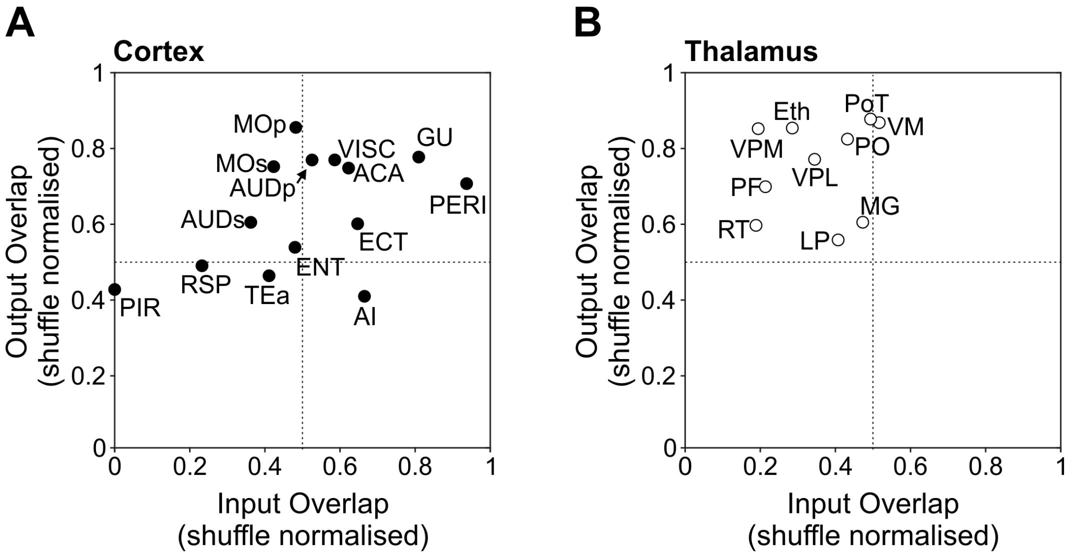

Supplementary Figure 7. Input and output overlap coefficients normalized by the mean of the shuffled distribution.

A, Input and output overlap coefficients normalized by the shuffle distribution mean for each cortical region. The position of the data points indicate similar amounts of input/output spatial overlap within cortical regions. Each closed circle denotes an individual brain region. **B**, Same as (**A**), except for thalamic sub-regions, with the position of data points encompassing the upper left quadrant, indicating that there is a greater different in their input/output spatial overlap. Open circles represent data from individual brain region.

Supplementary Table 1. Abbreviations for brain regions.

Brain Region	Abbreviation
Anterior amygdalar area	AAA
Anterior cingulate area	ACA
Nucleus accumbens	ACB
Agranular insular area	AI
Anterior pretectal nucleus	APN
Primary auditory area	AUDp
Supplemental auditory area (dorsal, posterior, ventral)	AUDs
Basolateral amygdalar nucleus	BLA
Basomedial amygdalar nucleus	BMA
Bed nuclei of the stria terminalis	BST
Central amygdalar nucleus	CEA
Claustrum	CLA
Dorsal cochlear nucleus	CN
Ventral cochlear nucleus	CN
Cortical amygdalar area	COA
Caudoputamen	CP
Cuneate nucleus	CU
Cuneiform nucleus	CUN
Ectorhinal area	ECT
Entorhinal area	ENT
Ethmoid nucleus of the thalamus	Eth
Fundus of striatum	FS
Globus pallidus internal and external segment	GP
Gigantocellular reticular nucleus	GRN

Gustatory areas	GU
Inferior colliculus	IC
Intermediate reticular nucleus	IRN
Lateral amygdalar nucleus	LA
Lateral posterior nucleus of the thalamus	LP
Medial amygdalar nucleus	MEA
Medial geniculate complex	MG
Primary motor area	MOp
Secondary motor area	MOs
Midbrain reticular nucleus	MRN
Magnocellular reticular nucleus	MARN
Nucleus of the solitary tract	NTS
Piriform-amygdalar area	PAA
Periaqueductal gray	PAG
Paragigantocellular reticular nucleus	PGRN
Parabrachial nucleus	PB
Perirhinal area	PERI
Parafascicular nucleus	PF
Pontine gray	PG
Piriform area	PIR
Posterior limiting nucleus of the thalamus	POL
Posterior complex of the thalamus	PO
Posterior triangular thalamic nucleus	PoT
Pontine reticular nucleus (including subregions)	PRN
Parvicellular reticular	PARN
Nucleus of reuniens	RE
Red nucleus	RN

Nucleus raphe (including subregions)	RPh
Retrosplenial area	RSP
Reticular nucleus of the thalamus	RT
Superior colliculus (including subregions)	SC
Suprageniculate nucleus	SGN
Substantia nigra (including subregions)	SN
Superior olivary complex	SOC
Spinal nucleus of the trigeminal (including subregions)	SPV
Primary somatosensory area	SSp
Supplemental somatosensory area	SSs
Temporal association areas	TEa
Ventral anterior-lateral complex of the thalamus	VAL
Facial motor nucleus	VII
Visceral area	VISC
Primary visual area	VISp
Supplemental visual area (anterolateral, rostrolateral, posteromedial, and anteromedial visual areas)	VISs
Ventral medial nucleus of the thalamus	VM
Vestibular nuclei (lateral, medial)	VNC
Ventral posterolateral nucleus of the thalamus including VPL parvicellular part	VPL
Ventral posteromedial nucleus of the thalamus including VPM parvicellular part	VPM
Zona incerta	ZI

Supplementary Table 2. Whole-brain inputs.

	fpIC ipsi mean (%)	fpIC ipsi SEM (%)	fpIC ipsi SD (%)	fpIC contra mean (%)	fpIC contra SEM (%)	fpIC contra SD (%)	fS1 ipsi mean (%)	fS1 ipsi SEM (%)	fS1 ipsi SD (%)	fS1 contra mean (%)	fS1 contra SEM (%)	fS1 contra SD (%)	ipsi t value	ipsi p value (fS1 vs. fpIC)	contra t value	contra p value (fS1 vs. fpIC)	# mice with label in fpIC	# mice with label in fS1
**AAA**	0.04	0.04	0.07	0	0	0	0	0	0	0	0	0	-1.08	0.310	0	0	2	0
**ACA**	0.03	0.01	0.03	0.11	0.07	0.13	0.29	0.2	0.4	0.03	0.02	0.04	1.30	0.231	-1.19	0.269	4	5
**AI**	0.8	0.13	0.25	0.21	0.05	0.1	0.35	0.09	0.18	0.13	0.04	0.07	-2.91	0.020	-1.34	0.217	5	5
**AUDp**	2.5	0.51	1.02	0.23	0.16	0.31	0.16	0.05	0.11	0	0	0	-4.56	0.002	-1.49	0.176	5	5
**AUDs**	5.54	1.31	2.63	5.36	1.45	2.91	0.97	0.35	0.69	0.03	0.01	0.03	-3.36	0.010	-3.66	0.006	5	5
**BLA**	0.06	0.04	0.07	0	0	0	0	0	0	0	0	0	-1.58	0.152	0	0	5	0
**BMA**	0.03	0.01	0.02	0	0	0	0	0	0	0	0	0	-2.41	0.042	0	0	4	0
**CEA**	0.02	0	0.01	0	0	0	0	0	0	0	0	0	-4.73	0.002	0	0	5	0
**COA**	0.05	0.04	0.08	0	0	0	0	0	0	0	0	0	-1.15	0.284	0	0	2	0
**ECT**	1.22	0.43	0.85	0.16	0.02	0.04	0.28	0.09	0.18	0.19	0.07	0.13	-2.14	0.064	0.55	0.597	5	5
**ENT**	0.02	0.02	0.03	0.07	0.03	0.05	0.02	0.01	0.02	0.04	0.01	0.02	0.02	0.982	-1.01	0.343	2	4
**Eth**	0.47	0.22	0.44	0	0	0	0.28	0.13	0.26	0	0	0	-0.77	0.465	0	0	4	4
**GU**	0.45	0.07	0.14	0.01	0	0.01	0.37	0.05	0.11	0.03	0.01	0.02	-0.92	0.386	1.96	0.086	5	5
**LA**	0.26	0.08	0.16	0	0	0	0	0	0	0	0	0	-3.27	0.011	0	0	5	0
**LP**	0.12	0.05	0.11	0.01	0	0.01	0.03	0.01	0.03	0.01	0.01	0.02	-1.54	0.162	0.77	0.466	4	3
**MEA**	0.04	0.02	0.04	0	0	0	0	0	0	0	0	0	-1.58	0.153	0	0	4	0
**MG**	0.5	0.1	0.21	0	0	0	0.03	0.01	0.03	0	0	0	-4.47	0.002	0	0	5	4
**MOp**	13.06	2.63	5.25	1.31	0.17	0.34	25.71	4.22	8.44	2.93	1.5	3	2.54	0.035	1.08	0.313	5	5
**MOs**	0.86	0.26	0.51	0.18	0.07	0.13	3.31	1.5	2.99	0.24	0.13	0.25	1.61	0.145	0.47	0.652	5	5
**PAA**	0.07	0.04	0.08	0	0	0	0	0	0	0	0	0	-1.65	0.138	0	0	5	0
**PERI**	0.04	0.01	0.03	0.02	0.01	0.02	0.01	0.01	0.01	0.04	0.01	0.02	-2.16	0.063	1.71	0.126	5	3
**PF**	0.03	0.01	0.03	0	0	0	0.06	0.03	0.05	0	0	0	0.85	0.418	-1.00	0.347	3	3
**PIR**	0.4	0.16	0.33	0.05	0.01	0.02	0.08	0.04	0.09	0.04	0.02	0.04	-1.91	0.093	-0.40	0.702	5	5
**PO**	0.41	0.13	0.26	0	0	0	2.59	0.74	1.49	0	0	0	2.89	0.020	-1.00	0.347	5	5
**POL**	0.18	0.08	0.16	0	0	0	0.01	0.01	0.01	0	0	0	-2.15	0.063	0	0	4	2
**PoT**	1.16	0.32	0.63	0	0	0	0	0	0	0	0	0	-3.67	0.006	-1.00	0.347	5	0
**RE**	0.22	0.06	0.13	0.08	0.05	0.09	0	0	0.01	0	0	0	-3.36	0.010	-1.66	0.135	5	1

**RSP**	0.38	0.09	0.17	0.12	0.07	0.14	0.14	0.04	0.07	0.07	0.03	0.06	-2.65	0.029	-0.71	0.498	5	4
**RT**	0.14	0.04	0.09	0	0	0	0.23	0.05	0.1	0.01	0.01	0.01	1.46	0.182	1.00	0.347	5	5
**SGN**	0.22	0.08	0.17	0	0	0	0	0	0	0	0	0	-2.59	0.032	0	0	5	0
**SSp**	35.68	2.9	5.81	4.86	0.8	1.6	28.69	0.98	1.97	4.18	1.25	2.49	-2.28	0.052	-0.45	0.662	5	5
**SSs**	7.72	0.95	1.91	4.73	1.04	2.09	19.67	3.44	6.87	0.22	0.05	0.1	3.35	0.010	-4.31	0.003	5	5
**TEa**	3.15	0.99	1.99	1.06	0.46	0.91	0.74	0.2	0.4	0.04	0.03	0.06	-2.38	0.045	-2.22	0.057	5	5
**VAL**	0.07	0.04	0.08	0	0	0	0.72	0.26	0.52	0.01	0.01	0.01	2.47	0.039	0.61	0.562	4	5
**VISC**	2.07	0.29	0.58	0.47	0.26	0.52	0.62	0.24	0.48	0.01	0.01	0.02	-3.84	0.005	-1.76	0.117	5	5
**VISp**	0.02	0.01	0.01	0.01	0.01	0.02	0.05	0.03	0.06	0.01	0.01	0.03	1.09	0.309	0.05	0.962	4	4
**VISs**	0.02	0.01	0.02	0.01	0.01	0.01	0.01	0.01	0.01	0	0	0	-0.58	0.577	-1.52	0.168	2	2
**VM**	1.06	0.3	0.6	0	0	0.01	0.49	0.15	0.3	0.01	0.01	0.01	-1.69	0.129	0.87	0.407	5	5
**VPL**	0.87	0.23	0.46	0	0	0	4.05	0.99	1.99	0.01	0.01	0.03	3.12	0.014	1.00	0.347	5	5
**VPM**	0.2	0.06	0.13	0	0	0	0.69	0.32	0.64	0.02	0.01	0.03	1.50	0.172	1.32	0.223	5	5
**ZI**	0.12	0.05	0.1	0.01	0.01	0.01	0.07	0.02	0.04	0.03	0.03	0.05	-1.05	0.326	0.75	0.476	4	5

Supplementary Table 3. Whole brain outputs.

	fpIC ipsi mean (%)	fpIC ipsi SEM (%)	fpIC ipsi SD (%)	fpIC contra mean (%)	fpIC contra SEM (%)	fpIC contra SD (%)	fS1 ipsi mean (%)	fS1 ipsi SEM (%)	fS1 ipsi SD (%)	fS1 contra mean (%)	fS1 contra SEM (%)	fS1 contra SD (%)	ipsi t value	ipsi p value (fS1 vs. fpIC)	contra t value	contra p value (fS1 vs. fpIC)	# mice with label in fpIC	# mice with label in fS1
**AAA**	0.05	0.01	0.02	0.01	0	0.01	0	0	0	0	0	0	-3.872	0.005	-2.700	0.027	5	0
**ACA**	0.42	0.06	0.12	0.12	0.03	0.05	0.77	0.31	0.62	0.1	0.02	0.03	1.080	0.312	-0.564	0.588	5	5
**ACB**	0.38	0.11	0.22	0.07	0.03	0.05	0.12	0.03	0.06	0.03	0.01	0.02	-2.220	0.057	-1.498	0.173	5	5
**AI**	1.65	0.39	0.79	0.34	0.07	0.14	0.45	0.12	0.23	0.22	0.05	0.11	-2.914	0.020	-1.441	0.188	5	5
**APN**	0.45	0.03	0.07	0.02	0	0.01	0.62	0.06	0.12	0.01	0.01	0.02	2.448	0.040	-0.620	0.553	5	5
**AUDp**	1.65	0.29	0.57	0.28	0.09	0.18	0.4	0.15	0.3	0.06	0.02	0.03	-3.876	0.005	-2.372	0.045	5	5
**AUDs**	3.57	0.44	0.89	1.12	0.23	0.47	1.76	0.3	0.6	0.36	0.05	0.09	-3.358	0.010	-3.224	0.012	5	5
**BLA**	0.22	0.07	0.14	0.03	0.01	0.02	0	0	0	0	0	0	-3.082	0.015	-2.482	0.038	5	0
**BMA**	0.09	0.03	0.05	0.03	0.01	0.02	0	0	0	0	0	0	-3.424	0.009	-2.447	0.040	5	0
**BST**	0.02	0.01	0.02	0.01	0.01	0.01	0	0	0	0	0	0	-2.484	0.038	-1.697	0.128	5	1
**CEA**	0.45	0.05	0.11	0.02	0.01	0.01	0	0	0	0	0	0	-8.244	0.000	-3.537	0.008	5	0
**CLA**	0.32	0.08	0.16	0.06	0.01	0.02	0.11	0.04	0.08	0.06	0.01	0.02	-2.369	0.045	-0.463	0.656	5	5
**CN**	0.05	0.02	0.04	0.03	0.01	0.02	0	0	0	0	0	0	-2.702	0.027	-3.414	0.009	5	0
**COA**	0.07	0.02	0.04	0.03	0	0.01	0	0	0	0	0	0	-3.139	0.014	-6.744	0.000	5	0
**CP**	13.27	1.31	2.62	1.51	0.29	0.59	14.72	0.94	1.88	5.25	0.28	0.57	0.901	0.394	9.159	0.000	5	5
**CU**	0	0	0	0.01	0.01	0.01	0	0	0	0.01	0.01	0.03	-0.967	0.362	0.531	0.610	1	0
**CUN**	0.12	0.02	0.03	0.01	0	0	0.03	0.01	0.01	0	0	0	-5.492	0.001	-4.515	0.002	5	5
**ECT**	1.6	0.13	0.25	0.32	0.06	0.11	0.7	0.09	0.18	0.28	0.04	0.08	-5.785	0.000	-0.572	0.583	5	5
**ENT**	0.84	0.24	0.47	0.13	0.03	0.07	0.18	0.05	0.1	0.11	0.04	0.07	-2.728	0.026	-0.326	0.753	5	5
**Eth**	0.44	0.06	0.11	0	0	0	0.35	0.03	0.05	0	0	0	-1.311	0.226	0.098	0.924	5	5
**FS**	0.09	0.03	0.07	0.04	0.02	0.04	0.01	0.01	0.01	0.01	0	0	-2.340	0.047	-1.684	0.131	5	2
**GP**	0.38	0.05	0.1	0.05	0.01	0.03	0.49	0.07	0.14	0	0	0	1.293	0.232	-3.579	0.007	5	5
**GRN**	0.13	0.03	0.06	0.3	0.04	0.08	0.27	0.12	0.25	0.44	0.17	0.34	1.082	0.311	0.782	0.457	5	5
**GU**	0.69	0.11	0.22	0.07	0.01	0.02	0.22	0.05	0.09	0.12	0.02	0.04	-4.015	0.004	2.511	0.036	5	5
**IC**	1.96	0.22	0.43	0.29	0.03	0.07	0.43	0.1	0.21	0.01	0.01	0.02	-6.416	0.000	-8.009	0.000	5	5
**IRN**	0.07	0.01	0.03	0.2	0.02	0.04	0.03	0.01	0.02	0.16	0.05	0.11	-2.167	0.062	-0.786	0.454	5	5

**LA**	0.14	0.05	0.1	0.03	0.01	0.02	0	0	0	0	0	0	-2.743	0.025	-3.237	0.012	5	0
**LP**	0.29	0.05	0.1	0.03	0.02	0.03	0.19	0.04	0.08	0	0	0	-1.514	0.169	-1.821	0.106	5	5
**MARN**	0.3	0.04	0.09	0.24	0.02	0.03	0.31	0.06	0.13	0.25	0.03	0.06	0.107	0.917	0.178	0.863	5	5
**MEA**	0.11	0.03	0.07	0.08	0.02	0.05	0	0	0	0	0	0	-3.503	0.008	-3.200	0.013	5	0
**MG**	0.83	0.16	0.31	0.02	0.02	0.03	0.2	0.03	0.06	0	0	0	-4.002	0.004	-1.364	0.210	5	5
**MOp**	4.69	0.86	1.72	0.34	0.04	0.09	9.22	1.5	2.99	2.95	0.31	0.61	2.625	0.030	8.436	0.000	5	5
**MOs**	1.65	0.36	0.72	0.11	0.02	0.04	4.64	0.72	1.44	0.35	0.09	0.18	3.710	0.006	2.605	0.031	5	5
**MRN**	5.04	0.54	1.08	0.32	0.04	0.08	3.39	0.55	1.11	0.14	0.09	0.19	-2.133	0.066	-1.792	0.111	5	5
**NTS**	0.02	0.01	0.01	0.04	0.02	0.03	0.01	0	0	0.02	0.01	0.02	-2.203	0.059	-0.674	0.520	5	4
**PAA**	0.06	0.02	0.03	0.02	0.01	0.02	0	0	0	0	0	0	-3.843	0.005	-2.228	0.057	5	0
**PAG**	2.84	0.25	0.49	0.42	0.08	0.15	0.85	0.34	0.67	0.14	0.1	0.2	-4.786	0.001	-2.228	0.057	5	5
**PARN**	0.05	0.01	0.02	0.18	0.03	0.07	0.03	0.01	0.03	0.11	0.04	0.08	-1.402	0.199	-1.213	0.260	5	5
**PB**	0.26	0.03	0.07	0.07	0.01	0.03	0.1	0.03	0.05	0.02	0.01	0.03	-3.635	0.007	-2.791	0.024	5	5
**PERI**	0.5	0.1	0.2	0.13	0.02	0.03	0.22	0.04	0.07	0.16	0.05	0.1	-2.722	0.026	0.467	0.653	5	5
**PF**	0.18	0.04	0.08	0.02	0.01	0.02	0.47	0.1	0.19	0.01	0.01	0.01	2.742	0.025	-0.506	0.626	5	5
**PG**	0.27	0.06	0.12	0.12	0.04	0.08	0.36	0.11	0.22	0.04	0.01	0.03	0.705	0.501	-1.923	0.091	5	5
**PGRN**	0.08	0.01	0.03	0.12	0.02	0.03	0.12	0.05	0.11	0.18	0.07	0.14	0.857	0.417	0.827	0.432	5	5
**PIR**	0.39	0.06	0.12	0.19	0.05	0.11	0.06	0.02	0.03	0.04	0.01	0.03	-5.418	0.001	-2.743	0.025	5	5
**PO**	0.89	0.13	0.25	0.01	0.01	0.01	1.49	0.14	0.28	0.02	0.01	0.02	3.167	0.013	1.109	0.300	5	5
**POL**	0.4	0.07	0.14	0.01	0	0	0.08	0.01	0.03	0	0	0	-4.495	0.002	-4.327	0.003	5	5
**PoT**	0.57	0.08	0.17	0.01	0	0	0	0	0	0	0	0	-6.814	0.000	-5.340	0.001	5	0
**PRN**	0.61	0.08	0.16	0.59	0.09	0.18	0.62	0.24	0.48	0.24	0.08	0.16	0.034	0.974	-2.807	0.023	5	5
**RE**	0.1	0.01	0.01	0.08	0.01	0.02	0.05	0.01	0.02	0.05	0.02	0.03	-4.750	0.001	-1.808	0.108	5	5
**RN**	0.13	0.02	0.04	0.02	0.01	0.01	0.44	0.13	0.26	0.01	0.01	0.01	2.404	0.043	-0.983	0.354	5	5
**RPh**	0.13	0.02	0.04	0.06	0.01	0.03	0.11	0.03	0.05	0.05	0.01	0.02	-0.701	0.503	-0.762	0.468	5	5
**RSP**	0.41	0.06	0.12	0.19	0.04	0.08	0.69	0.36	0.71	0.08	0.02	0.04	0.755	0.472	-2.441	0.041	5	5
**RT**	0.34	0.05	0.1	0.08	0.02	0.05	0.41	0.04	0.08	0	0	0	1.039	0.329	-3.525	0.008	5	5
**SC**	1.82	0.34	0.67	0.27	0.05	0.1	1.04	0.23	0.47	0.03	0.01	0.02	-1.901	0.094	-4.629	0.002	5	5
**SGN**	0.21	0.05	0.09	0	0	0	0.01	0	0.01	0	0	0	-4.138	0.003	-0.716	0.494	5	4
**SOC**	0.11	0.03	0.06	0.1	0.04	0.07	0.05	0.02	0.04	0.02	0.01	0.02	-1.613	0.146	-1.951	0.087	5	5
**SPV**	0.07	0.03	0.06	0.09	0.02	0.04	0.02	0.01	0.01	0.18	0.04	0.08	-1.608	0.147	1.996	0.081	5	5
**SSp**	15.44	1.21	2.42	1.39	0.17	0.34	15.04	1.26	2.51	6.81	0.71	1.43	-0.226	0.827	7.381	0.000	5	5

**SSs**	8.46	1.16	2.31	1.85	0.44	0.88	7.18	0.67	1.35	1.7	0.31	0.62	-0.956	0.367	-0.288	0.781	5	5
**TEa**	2	0.23	0.46	0.69	0.09	0.18	0.71	0.1	0.2	0.28	0.03	0.06	-5.131	0.001	-4.263	0.003	5	5
**VAL**	0.09	0.01	0.02	0	0	0	0.79	0.17	0.34	0.05	0.03	0.05	4.061	0.004	1.698	0.128	5	5
**VII**	0.06	0.02	0.03	0.16	0.02	0.04	0.02	0.01	0.01	0.08	0.02	0.03	-2.289	0.051	-3.089	0.015	5	5
**VISC**	1.9	0.16	0.32	0.59	0.17	0.34	0.89	0.08	0.16	0.55	0.16	0.31	-5.659	0.001	-0.159	0.877	5	5
**VISp**	0.23	0.05	0.1	0.06	0.02	0.04	0.1	0.05	0.1	0.02	0.01	0.01	-1.999	0.081	-1.924	0.091	5	5
**VISs**	0.13	0.06	0.11	0.02	0.01	0.03	0.08	0.05	0.1	0	0	0	-0.742	0.479	-0.994	0.350	5	5
**VM**	0.46	0.02	0.04	0.03	0	0.01	0.67	0.09	0.17	0.06	0.03	0.06	2.385	0.044	0.789	0.453	5	5
**VNC**	0.05	0.01	0.02	0.07	0.02	0.03	0.21	0.12	0.25	0.21	0.12	0.23	1.312	0.226	1.187	0.269	5	5
**VPL**	0.88	0.09	0.19	0.02	0.01	0.01	1.04	0.09	0.18	0.01	0	0.01	1.165	0.278	-2.168	0.062	5	5
**VPM**	0.94	0.08	0.16	0.06	0.02	0.03	1.14	0.11	0.23	0.01	0.01	0.01	1.465	0.181	-2.491	0.037	5	5
**ZI**	1.23	0.06	0.13	0.04	0.01	0.02	0.97	0.11	0.22	0.01	0	0.01	-1.991	0.082	-3.637	0.007	5	5

Supplementary Movie 1. Movie of 3D mouse brain showing whole brain inputs to fS1 (green) and fpIC (magenta) from *n* = 5 mice.

Supplementary Movie 2. Movie of 3D mouse brain showing whole brain outputs from fS1 (green) and fpIC (magenta) from *n* = 5 mice.

Supplementary References

Puchades, M.A., Csucs, G., Ledergerber, D., Leergaard, T.B., and Bjaalie, J.G. (2019). Spatial registration of serial microscopic brain images to three-dimensional reference atlases with the QuickNII tool. PLOS ONE *14*, e0216796. https://doi.org/10.1371/journal.pone.0216796.

Vestergaard, M., Carta, M., and Poulet, J.F.A. (2022). The cellular coding of temperature in the mammalian cortex. BioRxiv 2022.02.21.481299. https://doi.org/10.1101/2022.02.21.481299.

Wang, Q., Ding, S.-L., Li, Y., Royall, J., Feng, D., Lesnar, P., Graddis, N., Naeemi, M., Facer, B., Ho, A., et al. (2020). The Allen Mouse Brain Common Coordinate Framework: A 3D Reference Atlas. Cell *181*, 936-953.e20. https://doi.org/10.1016/j.cell.2020.04.007.
